# Supplemented Alkaline Phosphatase Supports the Immune Response in Patients Undergoing Cardiac Surgery: Clinical and Computational Evidence

**DOI:** 10.3389/fimmu.2018.02342

**Published:** 2018-10-11

**Authors:** Alva Presbitero, Emiliano Mancini, Ruud Brands, Valeria V. Krzhizhanovskaya, Peter M. A. Sloot

**Affiliations:** ^1^High Performance Computing Department, ITMO University, Saint Petersburg, Russia; ^2^Institute for Advanced Studies and Computational Science Laboratory, University of Amsterdam, Amsterdam, Netherlands; ^3^Complexity Institute, Nanyang Technological University, Singapore, Singapore; ^4^Alloksys Life Sciences BV, Wageningen, Netherlands

**Keywords:** alkaline phosphatase, innate immune response, cardiac surgery, ODE model, *in-silico*, clinical trial

## Abstract

Alkaline phosphatase (AP) is an enzyme that exhibits anti-inflammatory effects by dephosphorylating inflammation triggering moieties (ITMs) like bacterial lipopolysaccharides and extracellular nucleotides. AP administration aims to prevent and treat peri- and post-surgical ischemia reperfusion injury in cardiothoracic surgery patients. Recent studies reported that intravenous bolus administration and continuous infusion of AP in patients undergoing coronary artery bypass grafting with cardiac valve surgery induce an increased release of liver-type “tissue non-specific alkaline phosphatase” (TNAP) into the bloodstream. The release of liver-type TNAP into circulation could be the body's way of strengthening its defense against a massive ischemic insult. However, the underlying mechanism behind the induction of TNAP is still unclear. To obtain a deeper insight into the role of AP during surgery, we developed a mathematical model of systemic inflammation that clarifies the relation between supplemented AP and TNAP and describes a plausible induction mechanism of TNAP in patients undergoing cardiothoracic surgery. The model was validated against clinical data from patients treated with bovine Intestinal AP (bIAP treatment) or without AP (placebo treatment), in addition to standard care procedures. We performed additional *in-silico* experiments adding a secondary source of ITMs after surgery, as observed in some patients with complications, and predicted the response to different AP treatment regimens. Our results show a strong protective effect of supplemented AP for patients with complications. The model provides evidence of the existence of an induction mechanism of liver-type tissue non-specific alkaline phosphatase, triggered by the supplementation of AP in patients undergoing cardiac surgery. To the best of our knowledge this is the first time that a quantitative and validated numerical model of systemic inflammation under clinical treatment conditions is presented.

## Introduction

Alkaline Phosphatase (AP) is an enzyme originally known for its pivotal role in skeletal mineralization [1] but also for its capability to reduce inflammation. AP is in fact capable to reduce inflammation in animals by dephosphorylating inflammation triggering moieties like bacterial lipopolysaccharides (LPS) and extracellular nucleotides (2–9). In addition, several studies demonstrated that AP has a key function in maintenance and restoration of physiological barriers (10) in addition to this anti-inflammatory role of AP. In fact, many, if not all of these barriers may become hyper-permeable and or dysfunctional during such systemic ischemic and inflammation triggering insult. Extracellular nucleotides, like Adenosine Triphosphate (ATP) and Adenosine Diphosphate (ADP), having pivotal energy housekeeping functions, intracellularly act as ITM as soon they have leaked out of cells exposed to ischemic insults (11–13). LPS (14), a major component of the Gram negative bacterial outer membrane that is responsible for mediating septic shock (15). These inflammation triggering moieties (ITMs) are pro-inflammatory signals that may start local and systemic inflammatory responses in the innate immune system (16–18). Clinical trials involving the parenteral administration of AP to patients with severe sepsis, showed significant improvement in renal function (19, 20).

Humans have four distinct AP isozymes: tissue-nonspecific AP (liver/bone/kidney type AP), which is the most predominant circulating form of isozyme, intestinal-, placental-type, and germ cell AP. The anti-inflammatory effects of AP have been confirmed in settings with intestinal-, placental-, and liver-type AP.

Coronary artery bypass grafting (CABG) is one of the most common types of open-heart surgery which very often triggers a systemic inflammatory response, the clinical impact thereof is specific for the specific patient and depends on multiple factors like age, underlying diseases, and other confounding factors. The average annual number of CABG procedures in Western practice is about 62.2 per 100 000, ranging from 29.3 procedures in Spain to 135.4 procedures in Belgium (21). According to the Society of Thoracic Surgeons National Database, CABG-mediated complications contribute to 1.8% in-hospital and 2.2% operative mortalities, but caused 24% post-operative atrial fibrillation incidences in 151,474 patients in 2015 (22). We focus on the experiments by Kats et al. where we assume a systemic insult due to the amounts of ITMs introduced and generated peri- and post-cardiac surgery. Cardiac surgery invokes a vigorous systemic inflammatory response where massive amounts of ITMs are simultaneously generated from various sources in the body: (a) CABG and valve surgery under CPB (Cardio Pulmonary Bypass) induces sheer stress on blood cells damaging them and releasing a massive amount of ITMs in the process, (b) surgical area where tissue is damaged locally, and (c) reperfusion damage by accumulated ITMs that have crossed the gut barrier during hypo-perfusion and become systemically available upon re-circulation (23). The body thereby deals with a massive amount of ITMs that enter the circulation and are transported into the tissue via blood flow, and circulate in the blood stream due to the effects of cardio pulmonary bypass grafting and reperfusion injury.

*De novo* synthesis and release in circulation of AP induced by AP prophylaxis could be the body's way to improve its defense mechanism. A study in 2012 by Kats et al. (24) demonstrated that intravenous bolus administration and continuous infusion of bovine intestinal Alkaline Phosphatase [bIAP, bRESCAP, and APPIRED studies by Alloksys Life Sciences (7, 9)], in patients undergoing CABG (with or without valve surgery) results in the release of endogenous tissue non-specific AP (TNAP), most likely liver-type AP. This release exhibits a *unique* feat that was not before observed in septic shock patients (19). Induction of liver-type non-specific AP supports the idea that AP contributes significantly to the immune response. Additional Phase III clinical trials are currently on the way to confirm the beneficial effects of AP previously reported in CABG and valve surgery.

If indeed excess AP or the release of additional liver-type TNAP is beneficial to the clinical outcome of patients undergoing major surgeries as well as for individuals suffering from acute and chronic inflammation, then there is an urgent need to develop computational models that can reproduce and predict the dynamics of induced TNAP in circulation. The developed model could then pave way to better understand when and more importantly how much of this liver type TNAP is expressed and released back into circulation through *in-silico* experiments. We develop a new model of systemic inflammation based on existing models of the innate immune response to acute inflammation (25–32), with the purpose of describing, and gaining further insight on the dynamics of the innate immune system response through *in-silico* experiments (33). We thereby report, to the best of our knowledge, the first calibrated and validated mathematical model for systemic inflammation.

The human innate immune system (HIIS) is the body's first line of defense to an infection or trauma. This is commonly manifested in the form of acute inflammatory response, resulting from these and other oxidative stress conditions (34). Numerous studies were reported aiming to understand the acute inflammatory response based on the response of a single population of white blood cells to invading pathogens (25–29). Unlike in previous models that only deal with a general population of invading and invaded entities, Dunster et al. (30) distinguished between populations of white blood cells by incorporating activated macrophages, activated, apoptotic, and necrotic neutrophil populations. More specific mathematical models of HIIS that further distinguish white blood cells into distinct populations have also been developed. For instance, Su et al. (31) used a system of partial differential equations (PDE) that capture the spatial and temporal dynamics of the innate and adaptive immune response via the following stages: recognition, initiation, effector response, and resolution of infection. This model of the human innate immune response was adapted by Pigozzo et al. (32) who focused on the dynamics of LPS, neutrophils, pro-inflammatory, and anti-inflammatory cytokines.

This paper focuses on how the concentrations of the innate immune response components evolve over time. Partial differential equations (PDEs) provide ways to analyse both time and spatial dynamics of key aspects of HIIS. Since we are modeling a systemic insult, where a massive amount of ITMs are coming from various sources in the body and inflammation is not confined to a specific tissue or organ, we can assume that these moieties are present and distributed all throughout the organism. Thus, we regard the “tissue” as representative of the entire body. Given this assumption, the role of microscopic spatial effects for the dynamics of the system is negligible and we use ordinary differential equations (ODEs) to describe the dynamics of the immune response. However, we take into account spatial effects by modeling various compartments (liver, blood stream, and tissue) and the transport of cells and molecules between them due to the inflammation in blood and tissue. This compartmentalization of the organism allows us to account for chemotaxis at a macroscopic level using the change in permeability of the endothelium during the different stages of inflammation to affect the transport of immune cells and molecules between blood and tissue.

We therefore construct the HIIS model from Reynolds et al. (28), Su et al. (31), and Pigozzo et al. (32) by introducing the following key differences: compartmentalization of the organism into liver, blood and tissue; introduction of the dual pathway to neutrophils death, necrosis being pro-inflammatory and apoptosis being anti-inflammatory; introduction of the anti-inflammatory action of AP and of the mechanism of AP induction; the dilution of cellular components in tissue typical of systemic inflammatory responses as opposed to the increased concentration of cellular components in a localized region typical of acute inflammatory responses.

## Materials and methods

### Clinical trial data

Patients undergoing open-heart surgery were stratified according to a risk assessment score system called EuroSCORE (Type 1). EuroSCORE is a risk measure for severe complications (mortality) associated with this type of surgery. In addition to standard care treatment, patients undergoing cardiothoracic surgery were divided into two distinct categories based on the type of treatments they received: (a) placebo treatment (physiological buffer containing *no* AP) and (b) bovine intestinal AP (bIAP) treatment in the same buffer.

In the APPIRED I study patients with a 2 < EuroSCORE ≤ 6, were initially given either placebo or 1,000 IU of Bovine Intestinal AP (bIAP) followed by a continuous infusion during 36 h of either placebo (*n* = 31, with mean EuroSCORE = 3.7 ± 1.4) or 5.6 IU per kg body weight per hour (total 9,000 IU) (*n* = 32, with mean EuroSCORE = 3.6 ± 1.2).

In the APPIRED II study patients with a EuroSCORE of ≥ 5, were initially given either placebo (*n* = 25, with mean EuroSCORE 5.6 ± 2.6) or 1,000 IU of bIAP followed by a continuous infusion during 8 h (total 9,000 units) (*n* = 27 with mean EuroSCORE 5.8 ± 3.1) (total 9,000 units).

Where in APPIRED I, 63 patients underwent CABG only, in APPIRED II a total of 52 patients were included that underwent CABG combined with valvular surgery under CPB. This type of combined surgery is associated with an increased risk.

Further details of the APPIRED clinical trials have been described by Kats et al. (9). The induction of endogenous alkaline phosphatase in this trial was described in Kats et al. (24). Primary endpoints were cytokine levels peri- and post- surgery next to clinical outcome.

The APPIRED II data was used in sections Human Innate Immune System Model With the Induction Mechanism of TNAP 1 and HIIS Model Without the Induction Mechanism of TNAP 2 to validate the model. Data relative to APPIRED I was not used to validate the model due to the limited number of data points relative to AP. However, the secondary peak of ITMs observed in 16% of the patients in APPIRED I was used qualitatively to investigate the case of patients with complications in APPIRED II by adding a secondary source of ITMs *in-silico* in section Predicting The Innate Immune Response For Patients Having Excess ITMs Using Different AP Treatment Regimens. This secondary source of ITMs is relative to the documented median peak IL6 concentration in septic shock patients in Damas et al. (35).

Patients underwent CABG combined with valvular surgery. CABG pumps (heart-lung machine) were used during the surgery. The operations were primary, therefore operated specifically for open heart surgery, and were planned prior to the actual surgery, hence non-emergent. An intra-aortic balloon pump was not used during the surgery, except for one patient who exhibited cardiogenic shock with multi-organ failure. The surgery lasted for an average of 4.7 ± 1.4 h. The average perfusion time was 134 ± 40 min and average cross clamping time was 105 ± 40 min. Pre-medication such as relaxants, anesthetics, antibiotics, and blood products such as red blood cells or platelets were given prior and during the CABG surgery. We summarize patients' demographics, type and method of cardioplegia used, and patients' medical history in Tables 1–3 of the Supplementary Material respectively.

The data used was approved by the Ethics committee with IRB approval number M09-1965. The set-up of the study as well as appropriate consent procedures, have been reviewed and approved by the Institutional Review Board (METC). The central Independent Ethics Committee of The Netherlands (CCMO) has been informed. Approval from the METC of ZOL Genk was obtained in 6. March, 2012. The Belgium Competent Authority office: FAGG (Federaal Agentschap voor Geneesmiddelen en Gezondheidsproducten) was informed about the METC approval to initiate the study.

### Biological mechanism and model description

The HIIS model is constructed based on the biological mechanisms that occur in three separate compartments—blood, tissue, and liver. The blood and tissue are separated by the endothelial lining that acts as a modulated barrier toward accessing blood circulation derived components like immune cells. Models of acute inflammation commonly neglect the dynamics of immune cells in the blood compartment under the assumption that it plays a minor role on the dynamics of the innate immune response, acting as a reservoir of immune cells. Systemic inflammation is considerably different and it is characterized by a dilution of the immune cells in tissue caused by the delocalized inflammation. Additionally AP is known to act on ITM both in blood and in tissue. For this reason it is crucial to model both blood and tissue compartments to accurately capture the dynamic of resolution of a systemic inflammatory response.

#### Blood compartment

Upon a systemic insult cytokines are released by both tissue cells and immune cells in tissue, with different rates. Cytokines then migrate first toward the endothelial barrier, with which they interact changing its permeability to recruit more immune cells into the inflamed tissue, and then in part migrate into the bloodstream. Since we only have data about the concentration of cytokines in blood, our model describes the dynamics of cytokines (IL-6 and IL-10) in the bloodstream. The rates of cytokines production used in our model represent the rate with which cytokines reach the bloodstream after being secreted by macrophages and necrotic neutrophils in tissue. These rates thus take into account not only the secretion rate by each cell type in response to the inflammatory state (presence of ITMs) but also of the mechanism of transport from tissue to the bloodstream.

In our model we assume that AP is the only component of the immune system that interacts with ITMs in the bloodstream, forming ITM-AP complexes that are later removed in the liver by Kupffer cells. Similarly to immune cells, the transport of AP and ITMs from the bloodstream into the tissue is controlled by a permeability factor.

#### Tissue compartment

**Figure 1:**
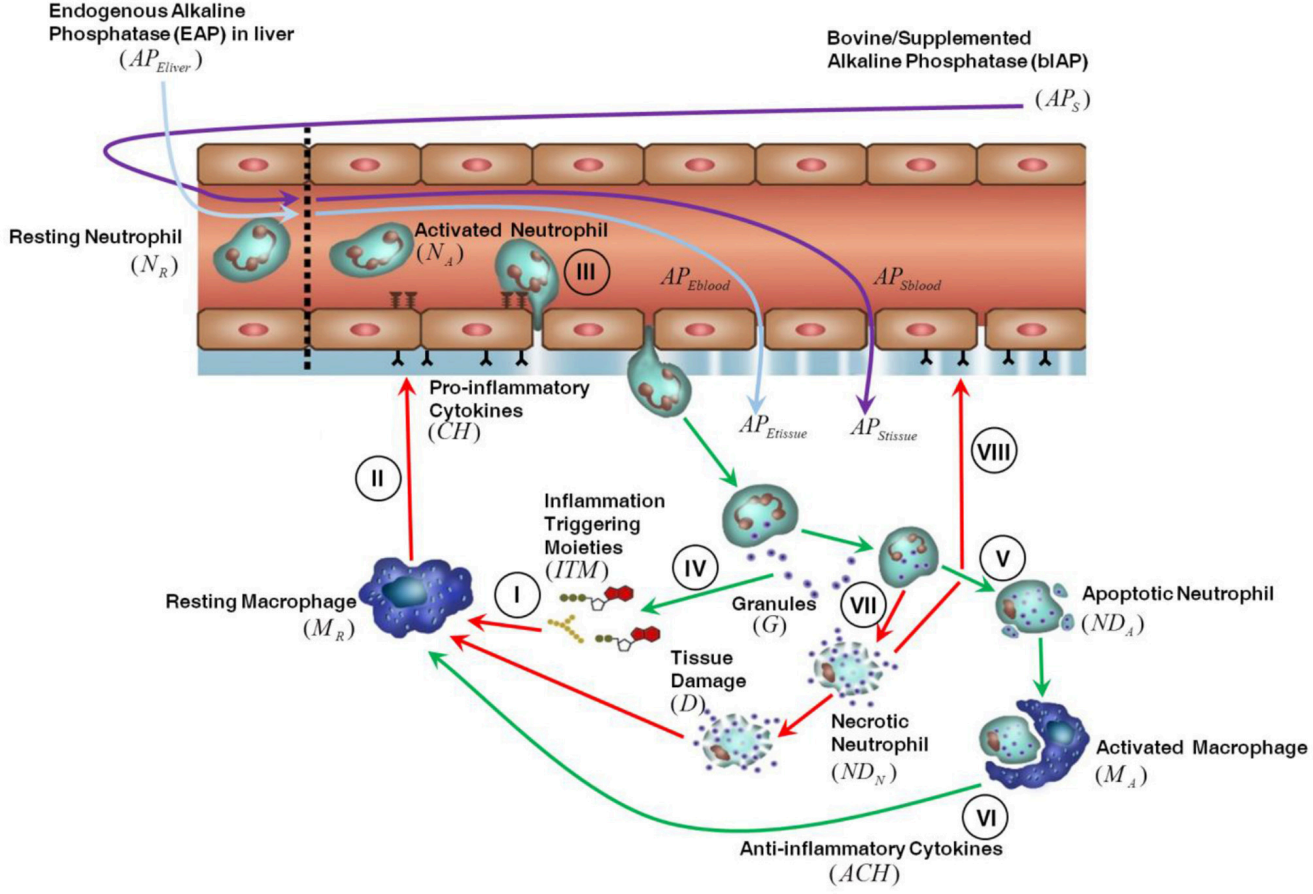
Description of the innate immune response to inflammation. For details see the main text.

The innate immune response triggered in tissue by invasive cardiac surgery is shown in Figure 1. We assume that the presence of ITMs in tissue is mostly due to the migration into the tissue of ITMs released in bulk in the circulation through damaged blood cells and gut hypo-perfusion and later transported via the bloodstream. ITMs in tissue are also due to local tissue damage caused by the invasive surgery, but we consider this amount negligible compared to the other two sources of ITMs. An inflammatory response is triggered as soon as ITMs activate resting macrophages (*M*_*R*_) leading them to differentiate into “activated” macrophages residing in tissue (*I*). Activated macrophages (*M*_*A*_) secrete pro-inflammatory cytokines (*CH*), which result in increasing the permeability of the endothelial barrier (*II*) via a series of intermediate stages. Consequently, resting neutrophils (*N*_*R*_) in circulation are primed by circulating ITMs and then enter the tissue through the endothelial barrier via a process called “diapedesis” (*III*). In the context of the computational model, resting neutrophils are only rendered active when they enter the tissue through the endothelial barrier. Activated neutrophils (*N*_*A*_) phagocytose and/or release their granules to neutralize or antagonize inflammation (*IV*). If the inflammation is cleared, the neutrophils go into apoptosis or programmed death (*V*). Activated macrophages remove the apoptotic neutrophils (*ND*_*A*_) by phagocytosis and in the process induce an anti-inflammatory effect as shown in Figure 1 by the green arrows (*VI*). If inflammation is too intense and not resolved rapidly, the neutrophils go into a necrotic (*ND*_*N*_) state (designated by the red arrows in Figure 1), which releases additional ITMs in the tissue (*VII*). The presence of yet another batch of ITMs in the tissue induces ongoing inflammatory responses that causes tissue damage, which in turn perpetuates overall inflammatory response by macrophage activation and neutrophil influx into the local inflamed tissue areas (*VIII*).

#### Liver compartment

At the onset of surgery a very high concentration of ITMs is released in the blood stream as a consequence of the damage to blood cells caused by the cardiac surgery bypass. In response to this massive ITM insult the liver releases all its stored AP (~5,300 IU) into the bloodstream. After 2–4 h the liver is able to supply newly synthesized AP again (36, 37). Endogenous AP is naturally produced by the body and is highly expressed at physiological barriers like the gut, placenta, lungs, kidney glomerulus, and the blood-brain barrier. Upon interaction with ITMs that are present in the bloodstream, endogenous AP is released from the apical membrane of specific physiological barriers expressing high levels of AP (like liver bile duct membrane, blood brain barrier, kidney, and gut) and brought into circulation or gut lumen as ITM-AP conjugates. ITM-AP complexes are eventually removed from circulation by the liver Kupffer cells (5). We assume that due to its size AP can, under normal non-inflammatory conditions, enter the tissue through the endothelial barrier fenestrae. Intravenously administered bovine AP (from here on noted as “bIAP treatment”) follows the same mechanism as endogenous AP, where it enters the tissue and detoxifies local ITMs through dephosphorylation. Note that the supplemented AP also detoxifies circulating ITMs directly and is removed from circulation by the Kupffer cells. In the case of an oxidative stress insult, such as that induced by cardiac surgery, ITM-AP is removed from circulation by Kupffer cells (5). This removal is observed in pre-clinical and clinical studies as a decrease in AP concentration in plasma. This decrease serves as a “*distress signal*” for the liver to release its stored AP at the bile ductal membrane barrier indirectly into the bloodstream (38). The release of liver AP into circulation implies that AP residing at blood- brain-, kidney-, and gut-barriers may also be released, compromising the integrity of these barriers. This may result in clinical phenotypic conditions like kidney failure and cognitive impairment observed upon major surgery. The hypothesis central to the AP intervention is that by replenishing AP through either *de novo* synthesis or supplementation during surgery, the impairments can be circumvented by helping reduce the inflammation and preserving the integrity of such barriers. *De novo* synthesis, in the strictest sense, refers to the general production of an entity. In the context of our model, we use the term “*de novo”* synthesis as the continuous production of AP by the liver.

Our HIIS model takes into account the following key mechanisms: (a) activation and inhibition of *M*_*R*_, (b) changes in endothelial permeability, (c) phagocytosis of rest products of ITMs, (d) phagocytosis of *ND*_*A*_ and *ND*_*N*_, (e) release of ITMs from necrotic cells, (f) natural death of immune cells and degradation of molecular entities, (g) production of *CH* and *ACH* (h), induction of *D*, and finally (i) delay in necrosis and cytokine production. The following key mechanisms are used to model the dynamics of AP: (a) release of endogenous/stored AP from the liver bile canalicular membrane, (b) *de novo* synthesis of AP in the liver, and (c) administration of bIAP into the bloodstream. The model does not take into account the AP released from other physiological barriers, since the amount of AP present on these is negligible compared to the AP released from the liver.

### Code implementation and repository

We used Python 3.6.5 on a 3.30 GHz Intel® Core™ i7-5820K CPU with 16.0 GB RAM in all our simulations. Python libraries used were: numpy, pandas, scipy, joblib, SALib, and scikit-learn. The python codes and sample data have been uploaded to https://github.com/avpresbitero/HIIS.

## Results

### Human innate immune system model with the induction mechanism of TNAP

In the first sub-section we describe first the calibration process of the model with data from the bIAP branch of APPIRED II under the assumption that supplemented bIAP stimulates the liver cells to produce additional TNAP. The calibrated model is then used to predict the dynamics of the immune response for the placebo branch. These predictions are validated using data from the placebo branch of APPIRED II. In the second sub-section we show the dynamics of all cellular and molecular entities in the model and highlight the action of bIAP on the dynamics of systemic inflammation.

In this study we use the median for each branch of the APPIRED II clinical trial to designate the values that best represent the population of patients undergoing cardiac surgery. Since we assume that the induction mechanism of alkaline phosphatase is inherent to all patients injected with bolus alkaline phosphatase, we did not cluster patients into different sub-groups. Although clustering patients into sub-groups based on their response would lead to a deeper understanding of the induction mechanism, the current dataset is not large enough to look into the individual trends of the patients' blood parameters. Providing a personalized take on the modeling of the innate immune response and on the individual response to the supplemented AP, this endeavor is beyond the scope of the current research but will be investigated in future studies.

#### Calibration and validation

**Figure 2:**
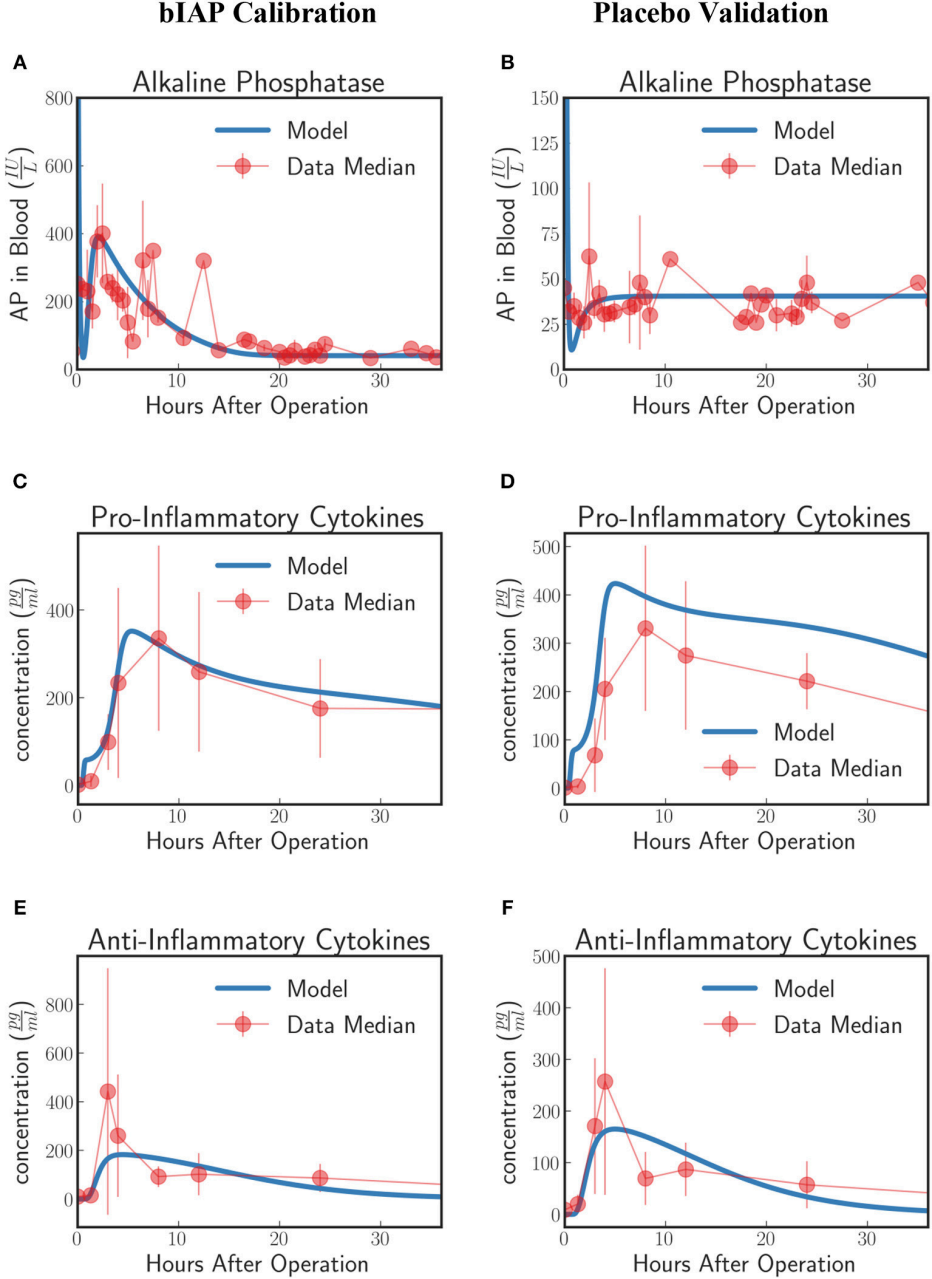
Innate immune response to systemic inflammation with the addition of the induction of TNAP by supplemented bIAP. The three plots **(A,C,E)** on the bIAP calibration column show the result of the calibration of the model parameters using data from the bIAP branch of APPIRED II. Data points are shown in red and correspond to the median value of the patients in this branch. The error bar shows the median absolute error. Blue lines correspond to the dynamics of the *in silico* model after calibration. **(A)** shows the dynamics of AP in blood **(B)** shows the dynamics of the pro-inflammatory cytokine represented in the model compared against IL6 data. **(C)** shows the dynamics of anti-inflammatory cytokines in the model against IL10 data. The three plots **(B,D,F)** on the placebo validation column show the validation of the model against data from the Placebo branch of APPIRED II. The model is able to predict the dynamics of placebo branch using the parameters calibrated with the data from the bIAP branch. The model predicts a protective effect of AP. As a consequence, the model predicts a greater concentration of pro-inflammatory cytokines in the placebo branch **(D)**. See unit conversion of AP and cytokines from $\frac{molecules}{mm^{3}}$ in Equations (32) and (33) (section 10 of the Supplementary Material) respectively.

Patients in the APPIRED studies were supplemented with a bolus of AP plus continuous infusion of AP and showed a surge of TNAP in the bloodstream (Figure 2A, bIAP Calibration). We calibrate the model parameters (summarized in 5 of the Supplementary Material) using three datasets: AP, pro-inflammatory, and anti-inflammatory cytokine profiles of patients in the bIAP treatment experiment. We summarize the results in Figure 2.

In response to a massive insult, the liver releases all its stored AP into the bloodstream. The liver then takes roughly 2 h to recover. We actually see this dynamics on the *in-silico* prediction of the model initially exhibited as a high concentration of AP at the onset of surgery. This is then followed by an immediate drop in AP concentration, corresponding to the time interval when the liver is still recuperating. Note that the effect of the AP bolus on the concentration disappears within 20 min after its supplementation as attributed to its short half-life and its interaction with ITMs. Then the liver begins supplying AP again at around 2 h after surgery.

A continuous supply of bIAP was administrated into the patients for 8 h, in addition to the initial concentration of 1,000 IU bovine AP. It was observed that liver-type TNAP is induced in these patients as is shown by the overall concentration of AP in circulation in Figure 2A. This supports the conjecture that, as a result of an ischemic condition, added AP serves as an indirect trigger for the liver to release more AP into the bloodstream. We therefore introduce an induction term $\frac{r_{inducepeak}}{1+\exp^{r_{induce}\left(t-t_{APdelay}\right)}}\;\left(AP_{stissue}+AP_{sblood}\right)$ in Equation (15) that is dependent on the concentration of bolus *AP* supplied into the system. The induction mechanism of AP is modeled as a reverse sigmoid function that is centered at 1 h—corresponding to the lag of release of AP from the liver, having flushed all its contents, as the liver recuperates.

The rate at which AP is being used up by the system and the rate at which AP is replenished back into the bloodstream from the liver should be the same regardless of the treatment type. This is because the two groups of patients (bIAP and placebo branches) underwent the same type of cardiothoracic surgical procedure. Hence, we assume the same scale of insult, or the same amount of ITMs on both branches. We validate our model by using the parameter values that we have previously calibrated on the supplemented AP treatment branch to predict the AP profiles of patients in the placebo treatment branch. Our results are shown in Figure 2B (Placebo Validation).

#### Dynamics of the HIIS with the induction mechanism of TNAP

##### Dynamics of macrophages.

**Figure 3:**
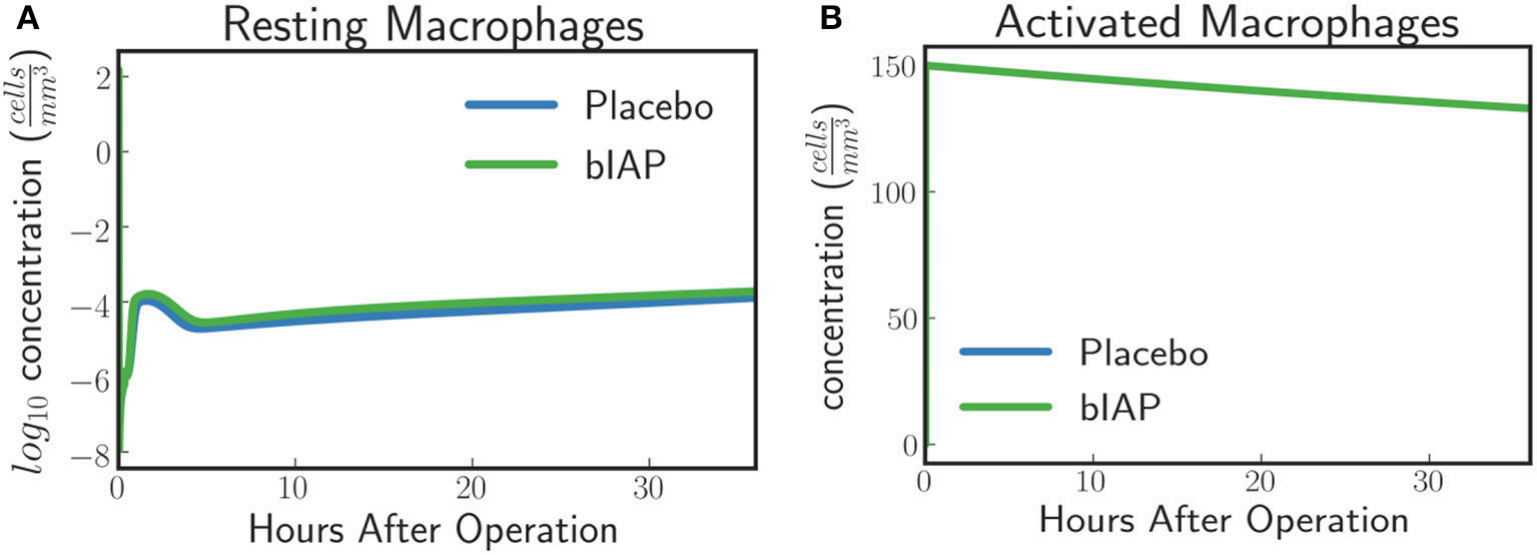
**(A)** Resting Macrophages residing in tissue concentration drops within very short time after initiating ischemic insult upon a massive insult and induced by circulating ITM released under CPB/surgical conditions, which in our case is cardiac surgery. **(B)** Activated Macrophages concentration is driven to a maximum brought about by the immediate turnover of resting macrophage population to activated macrophages.

In the case of a massive insult, such as cardiac surgery, where ITMs are simultaneously originating from numerous sources in the body, the entire population of resting macrophages immediately becomes activated. This is evident in Figure 3A where we see, from simulated data, an immediate drop of resting macrophage population at the moment surgery is initiated (time = 0), which corresponds to an immediate rise of activated macrophage population as shown in Figure 3B.

##### Dynamics of neutrophils.

In the context of our model, resting neutrophils become “*activated*” when they enter the tissue from the bloodstream via the endothelial barrier. The recruitment of neutrophils is proportional to the concentration of pro-inflammatory cytokines that increase the permeability of the endothelial barrier. This means that the larger the insult, the more resting neutrophils are recruited from the blood stream into the tissue.

**Figure 4:**
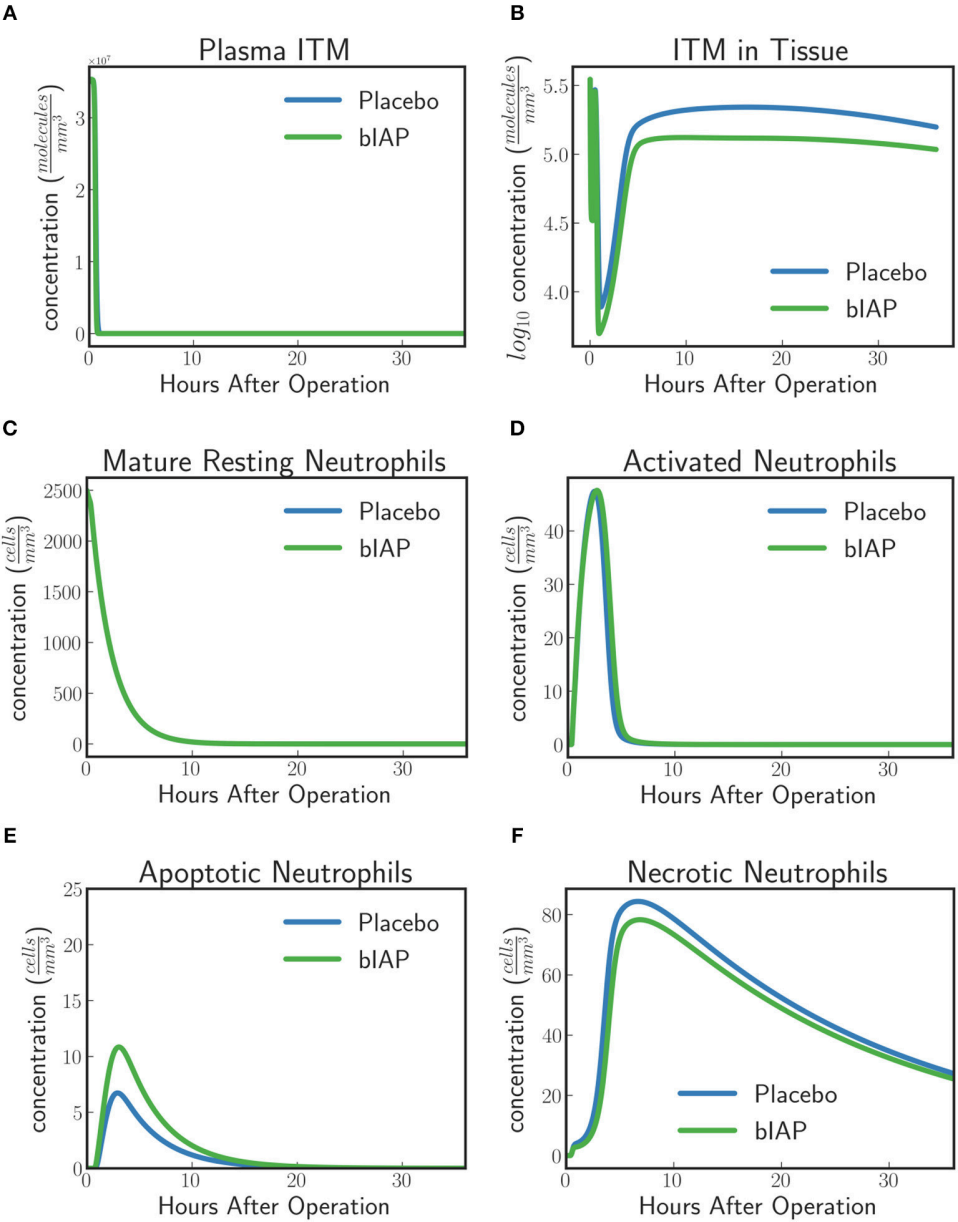
Dynamics of **(A)** ITMs in Plasma, **(B)** ITMs in Tissue, **(C)** Resting Neutrophils, **(D)** Activated Neutrophils, **(E)** Apoptotic Neutrophils, and **(F)** Necrotic Neutrophils in the tissue. Activated neutrophils in the placebo treatment go into necrosis quicker than in the supplemented AP treatment. It seems indeed that the addition of bolus AP contributes to the human innate immune system as an anti-inflammatory mediator by reducing the amount of neutrophils that go into the necrosis pathway.

For placebo patients the model predicts (Figure 4) an increased level of neutrophils necrosis in tissue in response to slightly higher concentration of ITM in tissue. The increased number of necrotic neutrophils leads to the production of additional ITMs, which acts as a positive feedback for inflammation. In AP-treated patients, the presence of additional AP prevents or reduces the necrosis of neutrophils. This could explain the lower number of adverse events reported for the AP branch of the clinical trial compared to the placebo branch (9).

The model dynamics shown in Figure 4 supports the idea that AP is an anti-inflammatory mediator that plays an important and active role in the human innate immune system, even though the impact of AP observed with the current levels of ITMs appears to be confined to a small shift in the ratio of apoptotic vs. necrotic neutrophils.

### HIIS model without the induction mechanism of TNAP

In this section we present the model results under the assumption that supplemented bIAP does not stimulate the liver cells to produce additional TNAP. In this case we use the previous model without the induction term introduced in section Calibration and Validation. We first attempted to calibrate the parameters of this alternative model on the supplemented bIAP branch. However, we were not able to model the AP dynamics of the bIAP branch without the induction term. For this reason we calibrated the model with data from the placebo branch of APPIRED II. The calibrated alternative model is then used to predict the dynamics of the immune response for the bIAP branch. We compare these predictions with data from the bIAP branch of APPIRED II and observe that the calibrated model fails to predict the AP dynamics observed in the clinical trial. Since the model without the induction mechanism cannot be validated we do not show the detailed dynamics of cellular and molecular entities as we did in section Dynamics of the HIIS With the Induction Mechanism of TNAP.

#### Calibration and validation

**Figure 5:**
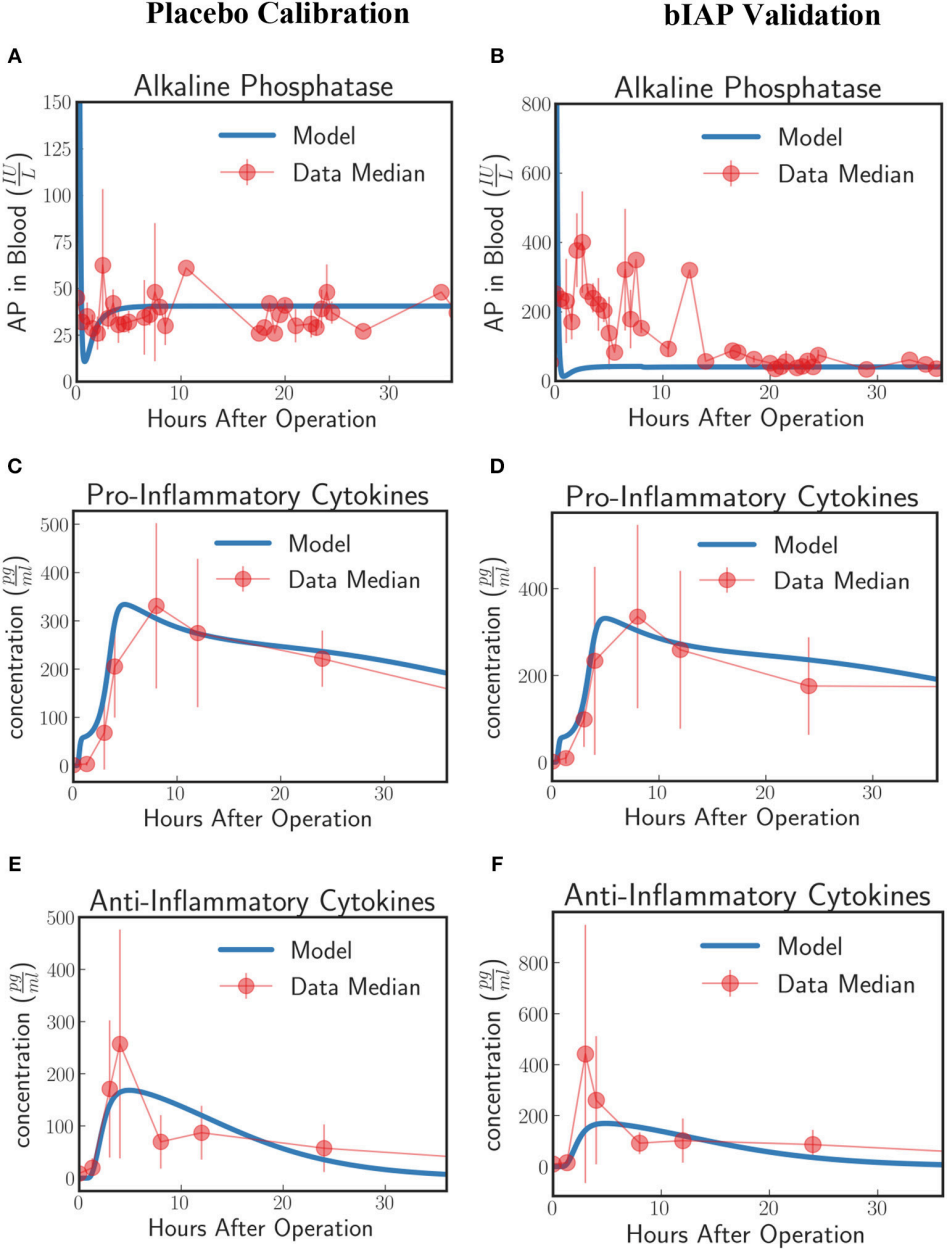
Innate immune response to systemic inflammation *without* the induction term. The three plots **(A,C,E)** on the placebo calibration column summarize the result of the calibration of the model parameters using data from the placebo branch of APPIRED II. Data points are shown in red and correspond to the median value of the patients in this branch. The error bar shows the median absolute error. Blue lines correspond to the dynamics of the *in silico* model after calibration. **(A)** shows the dynamics of AP in blood. **(B)** shows the dynamics of the pro-inflammatory cytokine represented in the model compared against IL6 data. **(C)** shows the dynamics of anti-inflammatory cytokines in the model against IL10 data. The three plots **(B,D,F)** on the bIAP validation column show the validation of the model against data from the bIAP branch of APPIRED II. Without the induction term, we are not able to reproduce the Alkaline Phosphatase profile in the AP-treated patients. See unit conversion of AP and cytokines from $\frac{molecules}{mm^{3}}$ in Equations (32) and (33) (section 10 of the Supplementary Material) respectively.

Instead of calibrating the parameters using the AP treatment and validating using the placebo treatment, we now reverse the process and calibrate instead the parameters in the placebo treatment first and validate them using the AP patient data. The aim of which is to find out whether we could model the induced amount of endogenous AP in the supplemented branch without using the induction term (see Figures 5A–F).

Using the calibrated parameters from the placebo study, we predict the dynamics of the bIAP study without the induction term. As shown in Figure 5B, the model without the induction term is not able to reproduce the AP profile of bIAP treatment patients. This clearly shows that an additional mechanism is missing and that the missing term has to account for additional production of TNAP to be released from the liver (the major source of stored AP) into the bloodstream. See Supplementary Material section 9 for details on the dynamics of the various compartments of the HIIS without the induction mechanism of TNAP.

### Predicting the innate immune response for patients having excess ITMs using different AP treatment regimens

The validation in section Human Innate Immune System Model With the Induction Mechanism of TNAP shows that the model with an induction mechanism for TNAP is able to predict the dynamics of the human innate immune system response in patients with systemic inflammation. Under the conditions of reported in APPIRED II patients in the placebo treatment branch have been able to resolve inflammation almost as effectively as patients in the bIAP branch, the only measurable difference being different levels of plasma AP and a reduced number of adverse events in the bIAP branch. Supplementation of AP under these conditions has an impact on the amount of necrotic neutrophils but did not drastically change the dynamics of immune cells from that of the placebo treatment group. However, since in the APPIRED I study 16% of patients show an excess amount of ITMs, the source of which is unknown, we explore the impact of supplemented AP in a system stressed by an additional source of ITMs after surgery. We model this scenario by adding a secondary source of insult *in-silico* and predicting how the human innate immune system would respond in different AP regimens using our model. The amount of this secondary source of ITMs is set to a reasonable value as indicated in Damas et al. (35). We perform two sets of *in-silico* experiments: in the first set we predict how the immune cells respond to an excess amount of ITMs; in the second set we predict how different AP regimens (i.e., different concentrations of bolus AP) affect the body's response to an additional source of ITMs as the one observed in APPIRED I.

#### In-silico experiment #1: innate immune system dynamics for patients with excess ITMs

**Figure 6:**
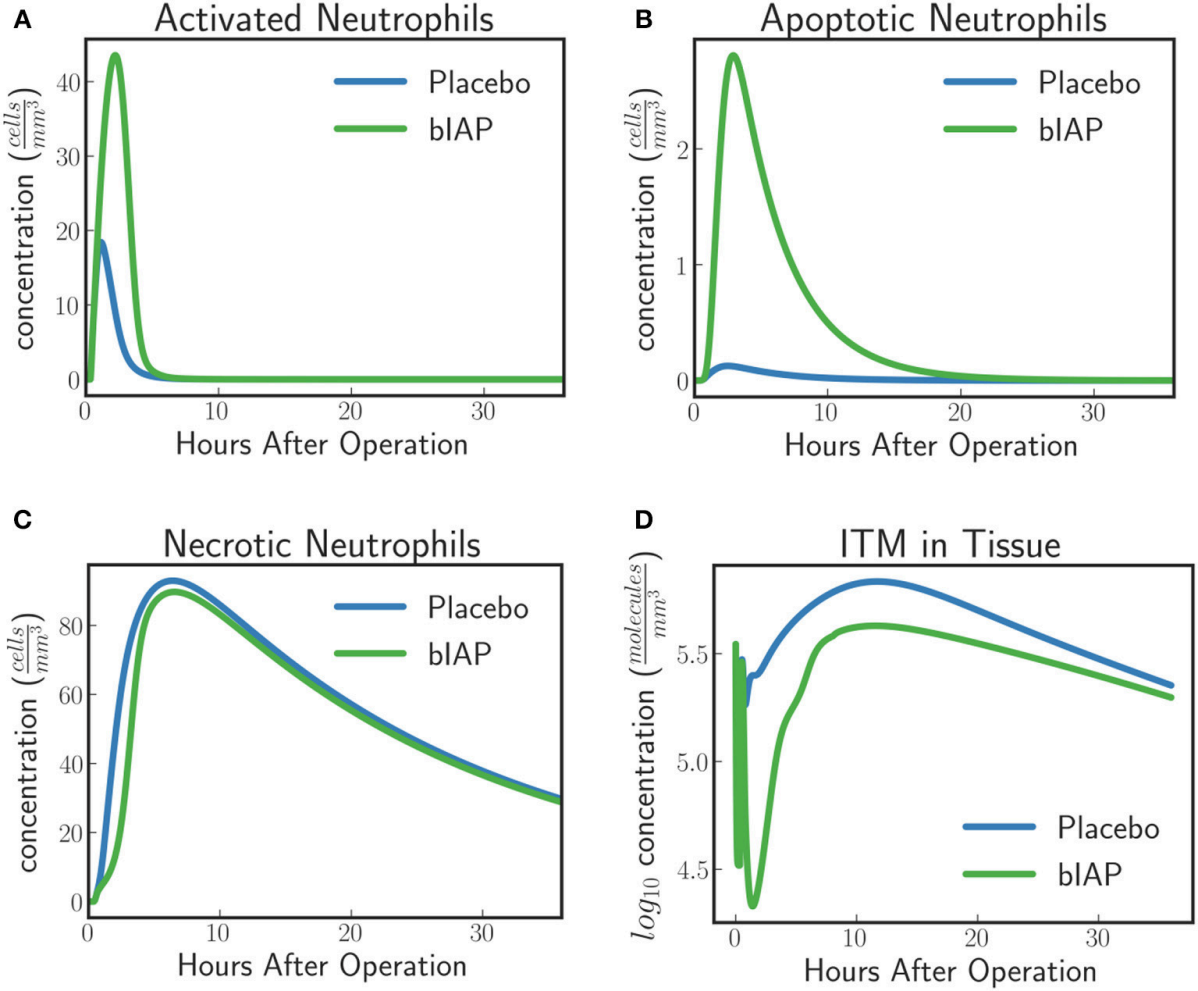
**(A)** Activated Neutrophils, **(B)** Apoptotic Neutrophils, **(C)** Necrotic Neutrophils, and **(D)** ITMs in Tissue in Supplemented and Placebo branches for patients with excess ITMs. The model predicts higher concentrations of apoptotic neutrophils in the supplemented than in the placebo branch, but higher concentrations of necrotic neutrophils in the placebo than in the supplemented branch.

**Figure 7:**
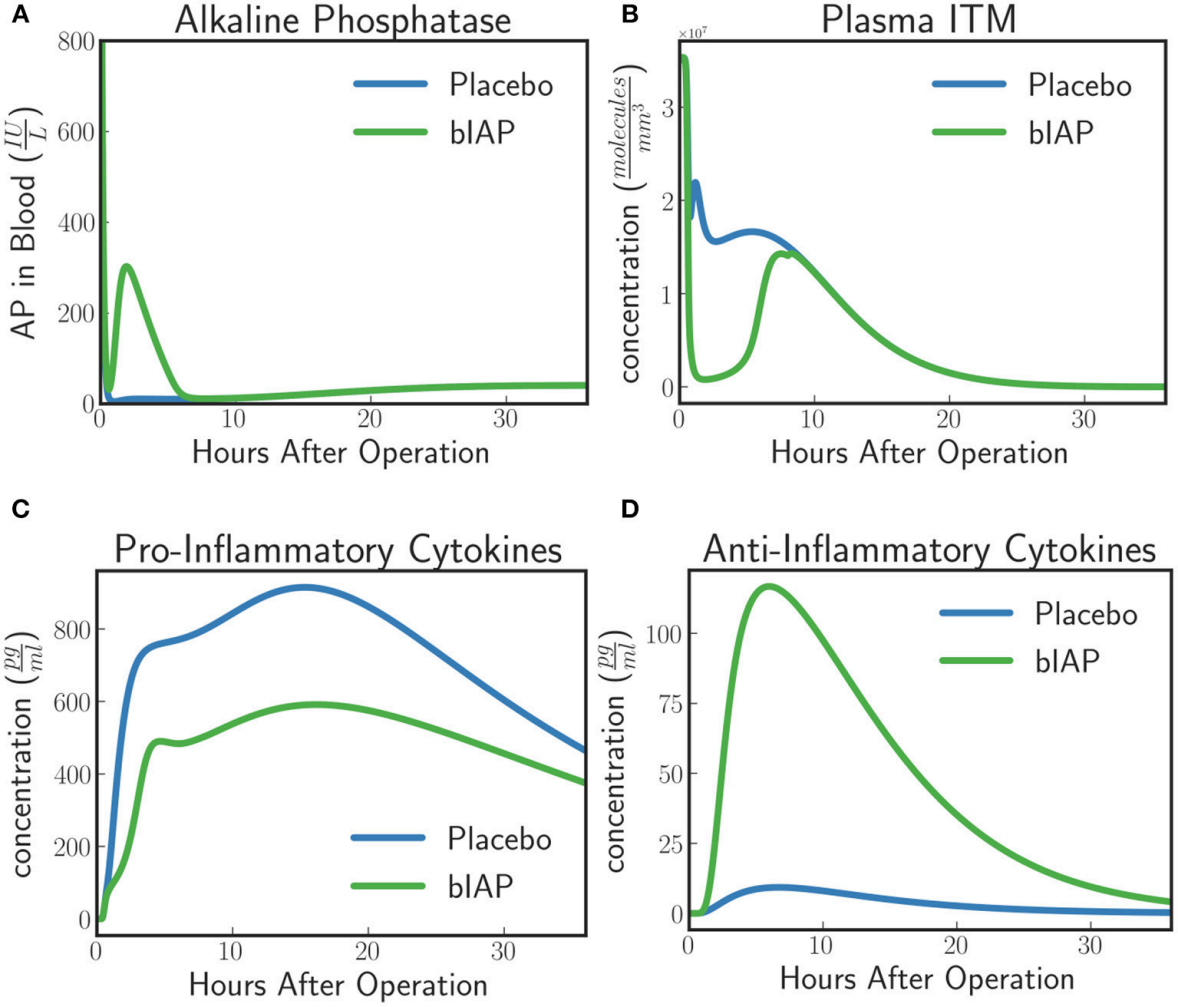
**(A)** Alkaline Phosphatase, **(B)** ITMs in Plasma, **(C)** Pro-inflammatory, and **(D)** Anti-inflammatory Cytokine Concentrations in Supplemented and Placebo branches for patients with excess ITMs. The placebo branch produces more pro-inflammatory cytokines and lesser anti-inflammatory cytokines, suggesting a more intense inflammation in the placebo branch compared to the supplemented branch. See unit conversion of AP and cytokines from $\frac{molecules}{mm^{3}}$ in Equations (32) and (33) (section 10 of the Supplementary Material) respectively.

The model predicts a protective effect due to supplemented AP when a patient is challenged by a second source of ITMs immediately after or during the surgery. Figures 6, 7 show the predicted dynamics for bIAP and placebo branches in case of a source of additional ITMs. Apoptotic neutrophils in supplemented branch have a concentration higher than in the placebo branch. On the other hand, necrotic neutrophils and ITMs in placebo branch are higher than in the supplemented treatment branch. The model predicts a more intense inflammation in the placebo branch as shown in the pro-inflammatory cytokines plot (Figure 7C). This is confirmed by (Figure 7D) which shows more anti-inflammatory cytokines in the supplemented than in the placebo branch.

#### In-silico experiment #2: treating patients with excess ITMs with various alkaline phosphatase regimen

Phase II and Phase IIIa clinical trials have observed an increased concentration of TNAP in circulation in the AP treatment group compared to the placebo group given an AP protocol. For instance in APPIRED I, a bolus of 1,000 IU of bovine AP was first injected to patients undergoing cardiac surgery followed by a continuous infusion of 5.6 IU/L per kg body weight for 8 h. In this section we predict the dynamics of the innate immune response under the conditions described in section *in-silico* Experiment #1: Innate Immune System Dynamics for Patients With Excess ITMs given two different AP supplementation regimens for which we increase the supplemented AP to twice and thrice the original protocol respectively. We then compare the results with the protocol tested in APPIRED II study.

**Figure 8:**
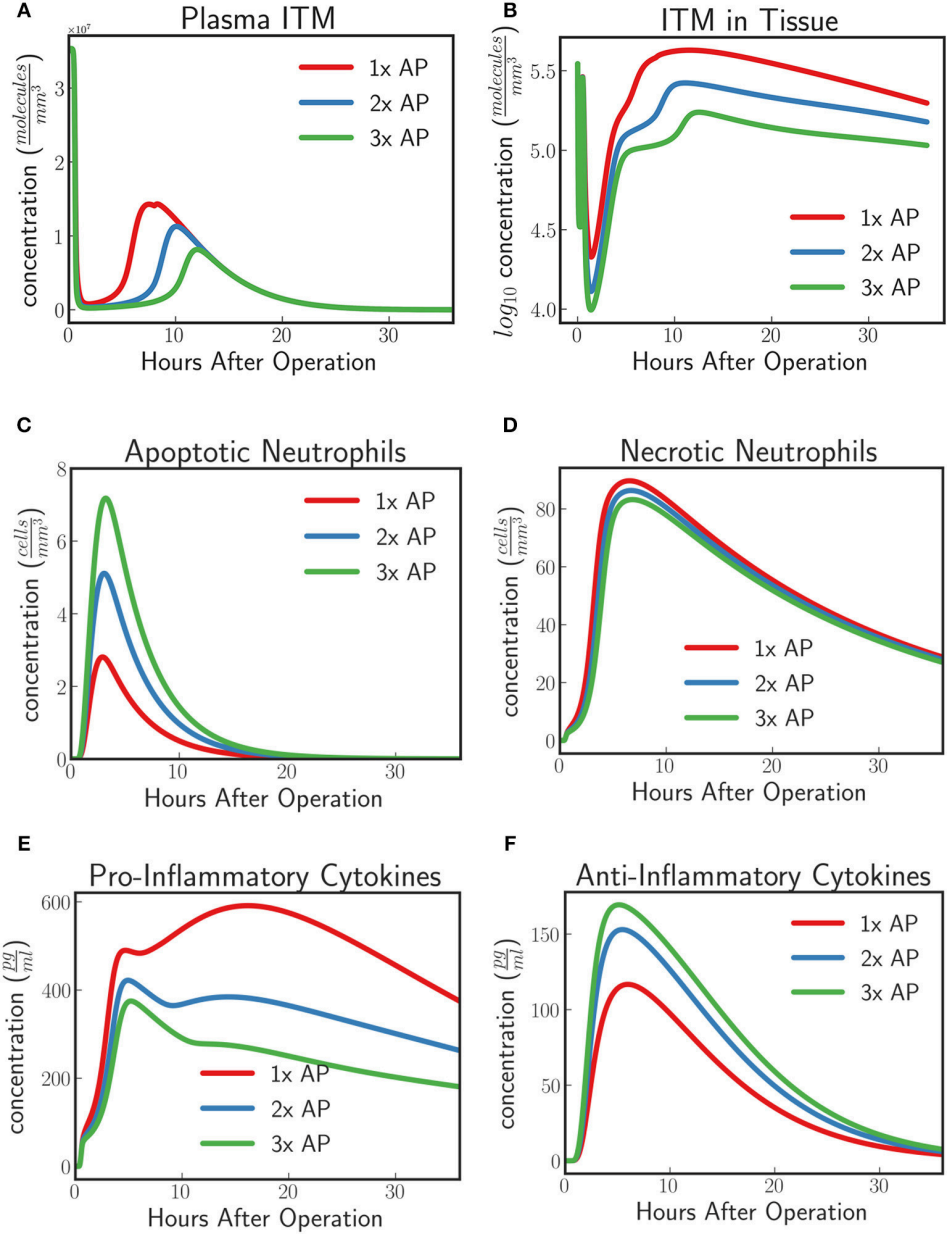
**(A)** ITMs in Plasma, **(B)** ITMs in Tissue, **(C)** Apoptotic Neutrophils, **(D)** Necrotic Neutrophils, **(E)** Pro-Inflammatory Cytokines, and **(F)** Anti-Inflammatory Cytokines for AP protocol APPIRED II (*red*) 2x the amount of AP, (*blue*), and 3x the amount of AP (*green*). Here we shoe that the model predicts an increasing protective effect the higher the concentration of supplemented AP by showing an increasing neutralizing effect on ITMs both in plasma and in tissue, increasing concentrations of apoptotic neutrophils and anti-inflammatory cytokines, and decreasing concentrations of necrotic neutrophils and pro-inflammatory cytokines. See unit conversion of cytokines from $\frac{molecules}{mm^{3}}$ in Equation (33) of section 10 of the Supplementary Material.

The model predicts an increasing protective effect the higher the concentration of supplemented AP by showing an increasing neutralizing effect on ITMs both in plasma and in tissue (Figures 8A,B). As the concentration of supplemented AP increases, more and more activated neutrophils are inclined to go into apoptosis rather than necrosis (Figures 8C,D). Pro-inflammatory profiles show that increasing supplemented AP decreases the amount of pro-inflammatory cytokines, while increasing the population of anti-inflammatory cytokines, indicating a better resolution of the systemic inflammation.

## Discussion

The induction mechanism of liver-type Tissue Non-specific Alkaline Phosphatase (TNAP) into circulation observed in patients undergoing coronary artery bypass grafting with cardiac valve replacement could be the body's way of strengthening its defense mechanism against such a massive insult, making TNAP a *de facto* key player in the human innate immune system. Hence, directing the attention to the role of Alkaline Phosphatase in systemic inflammation and understanding its role in supporting an appropriate innate immune response is of utmost immunological importance. Computational modeling makes it possible to mimic and understand such intricate details and mechanisms of the human innate immune system and offers a predictive power for experimental outcomes through *in-silico* experiments.

To our knowledge, we have developed the first mathematical model of systemic inflammation that is calibrated and validated on clinical data. We show that the amount of additional endogenous TNAP released in circulation in the supplemented branch is proportional to the total concentration of bolus AP being supplied. We also provide a plausible mathematical function that describes this mechanism. Our model predicts a protective effect of AP in the supplemented branch of APPIRED II, evidence of which can be concluded from the dynamics of the neutrophils. This effect, minimal under the conditions of APPIRED II, becomes obvious when patients exhibit an excess of ITMs after surgery (as observed in 16% of patients of APPIRED I study). We present a scenario where we mimic excess ITMs as seen in some patients by adding a secondary source of insult and see how the model reacts to different AP regimens by varying doses of AP supplied to patients. We show that additional AP has indeed a protective effect and this effect is more prominent in patients with excess ITMs. In this case *in-silico* experiments predict that the amount of apoptotic neutrophils in the supplemented AP branch is much higher than in the placebo branch. Additionally the amount of pro-inflammatory cytokines predicted for the supplemented AP branch is lower than in the placebo branch, giving further evidence that supplemented AP reduces the intensity of systemic inflammation. As expected the model predicts more anti-inflammatory cytokines in the supplemented branch than in the placebo branch. In other words, the dynamics predicted by the *in-silico* model show that resolution of inflammation is faster and more efficient with increasing concentration of AP. Hence, our findings suggest that AP indeed plays an important role in mitigating inflammation especially in systemic inflammation and that this protective effect can be modified through variation in AP protocol.

Our model for systemic inflammation is in fact similar to mechanistic models of physiological process, more specifically that of pharmacokinetic/pharmacodynamic (PKPD) models, that are used to develop insights on the dynamics and magnitude of the effect of a drug through quantitative analysis. These models are used to describe the dynamics of the physiological variables in different states. In our case, we model and validate the dynamics of bolus AP together with the human innate immune response for patients undergoing cardiac surgery in two treatment arms respectively: with or without AP supplementation. After validation, the model is used to understand and predict the effect of experimental perturbations, such as variations in AP regime, an approach that is referred to as “forward engineering.” A synergy, henceforth, is created between systems biology and PKPD through iterations between computational/mathematical modeling and experimentation (39). Helmlinger et al. have provided a comprehensive review of drug-disease modeling in the pharmaceutical industry (40). A robust application of systems pharmacology, or the application of systems biology in order to understand how drugs affect the human body, is detailed by Gadkar et al. (41).

The current model has three main limitations: the limited biological knowledge regarding the mechanism that regulates the induction of endogenous AP production triggered by supplemented AP, the potential bias introduced by the modeling approach, and the bias introduced by studying the median dynamics rather than attempting to cluster patients in groups with different dynamics.

1. The goal of this model is to prove the existence of AP induction and propose a possible mechanism describing this dynamics. We did validate the existence of the induction of AP, yet the available experimental data is not sufficient to unequivocally unravel the underlying mechanism.
2. The other limitation relates to the modeling approach used. Ordinary differential equations do not take into account the spatial properties of the innate immune response during systemic inflammation. The spatial effects are limited to the compartmentalization of the body into blood, liver and tissue while microscopic spatial effects of the cellular dynamics are neglected. It is possible that modeling the spatial properties of the system would result in a more accurate description of the dynamics, especially for the first 3 h during which there is a major displacement of cells between blood and tissue. For that, we would need a high resolution spatial-temporal clinical data.
3. Given the data at our disposal we decided to study the systemic inflammation via the median dynamics of the two branches of the APPIRED II clinical trial. The large variability in the dataset suggests that clustering patients in subgroups with different dynamics might provide a more accurate prediction of model parameters. However, we believe that the approach used in this paper is sufficient to prove the existence of the induction mechanism of AP and to provide a preliminary description of its dynamics.

The physiological relevance of this study is that to the best of our knowledge this is the first mathematical model describing systemic inflammation. Additionally this is the first model describing the role of Alkaline Phosphatase in the resolution of inflammation after invasive cardiac surgery, laying the foundations to understand systemic inflammatory response syndrome. The main clinically relevant result is the evidence of the existence of an induction mechanism triggered by supplemented AP. This model provides a starting point to investigate the amount of endogenous AP induced in the body, and consequently, the optimal amount of supplemented AP to be administered during and after invasive cardiac surgery.

Using the proposed model, we have shown *in-silico* the dynamics of systemic inflammation within a period of 36 h using different AP regimens. This information is being taken into account in the planning of a clinical trial phase III b. Data from the new multi-center clinical trial will be used to further refine the model. This model and its future iterations will be useful to predict the dynamics of neutrophils during systemic inflammation (namely the balance between apoptotic and necrotic neutrophils and the subsequent resolution of inflammation) and act as a tool to optimize the administration of anti-inflammatory drugs (not necessarily AP) in clinical trials dealing with systemic inflammatory response syndromes.

We perform a global sensitivity test (see Supplementary Material section 8) where we vary our input parameters within intervals that correspond to the values found in literature.

The model provides evidence of the existence of an induction mechanism of liver-type tissue non-specific alkaline phosphatase, triggered by the supplementation of AP in patients undergoing cardiac surgery. We show that the AP branch of the clinical trial can only be explained using a mechanism that induces a release in circulation of liver TNAP that is proportional to the amount of supplemented AP. We provide a possible mathematical description of this induction mechanism. The model is validated using novel clinical AP, pro-inflammatory and anti-inflammatory cytokine profiles of placebo- and bIAP-treated patients. This is the first time that liver-type tissue non-specific alkaline phosphatase has been modeled together with the human innate immune system. To date, there are no other existing published clinical trials that tackle the exact mechanisms of liver-type TNAP induction, let alone, a model that describes systemic inflammation. To the best of our knowledge this is the first numerical model of a complex innate immune response that is quantitatively validated with clinical data. Our work paves the way to a deeper understanding of the immunological mechanisms underpinning this important innate immune response to oxidative stress mediated inflammation.

## Ethics statement

The clinical trial data used in this study was carried out in accordance with the recommendations of Institutional Review Board (METC) and the central Independent Ethics Committee of The Netherlands (CCMO) with written informed consent from all subjects. All subjects gave written informed consent in accordance with the Declaration of Helsinki. The protocol was approved by the institutional review board (METC) and the central Independent Ethics Committee of The Netherlands (CCMO) has been informed. Approval from Belgium was from the METC of ZOL Genk and the Belgium Competent Authority office: FAGG (Federaal Agentschap voor Geneesmiddelen en Gezondheidsproducten) was informed about the METC approval.

## Author contributions

All authors have contributed substantially to the conception and design of the work. All authors have drafted and revised the work for intellectual content. All authors have equally provided the approval for plausible publication of the content. All authors have agreed to be accountable for all aspects of the work, which includes ensuring the accuracy and integrity of all parts of the work.

### Conflict of interest statement

RB was employed by company Alloksys Life Sciences BV. The remaining authors declare that the research was conducted in the absence of any commercial or financial relationships that could be construed as a potential conflict of interest.

## References

[1] Robison R. The possible significance of hexosephosphoric esters in ossification. Biochem J. (1923) 17:286–93. 16743183 10.1042/bj0170286 PMC1259346

[2] Poelstra K Bakker WW Klok PA Hardonk MJ Meijer DK. A physiologic function for alkaline phosphatase: endotoxin detoxification. Lab Invest. (1997) 76:319–27. 9121115

[3] Poelstra K Bakker WW Klok PA Kamps JA Hardonk MJ Meijer DK. Dephosphorylation of endotoxin by alkaline phosphatase *in vivo*. Am J Pathol. (1997) 151:1163–9. 9327750 PMC1858034

[4] Koyama I Matsunaga T Harada T Hokari S Komoda T. Alkaline phosphatases reduce toxicity of lipopolysaccharides *in vivo* and *in vitro* through dephosphorylation. Clin Biochem. (2002) 35:455–61. 10.1016/S0009-9120(02)00330-2 12413606

[5] Bentala H Verweij WR Huizinga-Van der Vlag A van Loenen-Weemaes AM Meijer DKF Poelstra K. Removal of phosphate from lipid A as a strategy to detoxify lipopolysaccharide. Shock (2002) 18:561–6. 10.1097/01.shk.0000043623.17707.47 12462566

[6] Beumer C Wulferink M Raaben W Fiechter D Brands R Seinen W. Calf intestinal alkaline phosphatase, a novel therapeutic drug for lipopolysaccharide (LPS)-mediated diseases, attenuates LPS toxicity in mice and piglets. J Pharmacol Exp Ther. (2003) 307:737–44. 10.1124/jpet.103.056606 12970380

[7] Van Veen SQ Dinant S Van Vliet AK Van Gulik TM Alkaline phosphatase reduces hepatic and pulmonary injury in liver ischaemia-reperfusion combined with partial resection. Br J Surg. (2006) 93:448–56. 10.1002/bjs.5275 16491472

[8] Goldberg RF Austen WG Zhang X Munene G Mostafa G Biswas S. Intestinal alkaline phosphatase is a gut mucosal defense factor maintained by enteral nutrition. Proc Natl Acad Sci USA. (2008) 105:3551–6. 10.1073/pnas.0712140105 18292227 PMC2265168

[9] Kats S Brands R Seinen W de Jager W Bekker MWA Hamad MA. Anti-inflammatory effects of alkaline phosphatase in coronary artery bypass surgery with cardiopulmonary bypass. Recent Pat Inflamm Allergy Drug Discov. (2009) 3:214–20. 10.2174/187221309789257388 19534671

[10] Kaliannan K Hamarneh SR Economopoulos KP Nasrin Alam S Moaven O Patel P. Intestinal alkaline phosphatase prevents metabolic syndrome in mice. Proc Natl Acad Sci USA. (2013) 110:7003–8. 10.1073/pnas.1220180110 23569246 PMC3637741

[11] Huizinga R Kreft KL Onderwater S Boonstra JG Brands R Hintzen RQ. Endotoxin- and ATP-neutralizing activity of alkaline phosphatase as a strategy to limit neuroinflammation. J Neuroinflammation (2012) 9:754. 10.1186/1742-2094-9-266 23231745 PMC3538711

[12] Moss AK Hamarneh SR Mohamed MMR Ramasamy S Yammine H Patel P. Intestinal alkaline phosphatase inhibits the proinflammatory nucleotide uridine diphosphate. AJP Gastrointest Liver Physiol. (2013) 304:G597–604. 10.1152/ajpgi.00455.2012 23306083 PMC3602687

[13] Malo MS Alam SN Mostafa G Zeller SJ Johnson P V Mohammad N. Intestinal alkaline phosphatase preserves the normal homeostasis of gut microbiota. Gut (2010) 59:1476–84. 10.1136/gut.2010.211706 20947883

[14] Poelstra K Bakker WW Klok PA Hardonk MJ Meijer DK. A physiologic function for alkaline phosphatase: endotoxin detoxification. Lab Invest. (1997) 76:319–27. 9121115

[15] Geddes K Philpott DJ. A new role for intestinal alkaline phosphatase in gut barrier maintenance. Gastroenterology (2008) 135:8–12. 10.1053/j.gastro.2008.06.006 18549817

[16] Matzinger P An innate sense of danger The signals that initiate immune responses. Semin IMMU Nol. (1998) 10:399–415. 10.1006/smim.1998.0143 9840976

[17] Miller SI Ernst RK Bader MW. LPS, TLR4 and infectious disease diversity. Nat Rev Microbiol. (2005) 3:36–46. 10.1038/nrmicro1068 15608698

[18] Riggle KM Rentea RM Welak SR Pritchard KA Oldham KT Gourlay DM. Intestinal alkaline phosphatase prevents the systemic inflammatory response associated with necrotizing enterocolitis. J Surg Res. (2013) 180:21–6. 10.1016/j.jss.2012.10.042 23158403 PMC5664146

[19] Pickkers P Snellen F Rogiers P Bakker J Jorens P Meulenbelt J. Clinical pharmacology of exogenously administered alkaline phosphatase. Eur J Clin Pharmacol. (2009) 65:393–402. 10.1007/s00228-008-0591-6 19048243

[20] Heemskerk S Masereeuw R Moesker O Bouw MP van der Hoeven JG Peters WH Alkaline phosphatase treatment improves renal function in severe sepsis or septic shock patients. Crit Care Med. (2009) 37:417–23 e1. 10.1097/CCM.0b013e31819598af 19114895

[21] Head SJ Kieser TM Falk V Huysmans HA Kappetein AP. Coronary artery bypass grafting: Part 1 - the evolution over the first 50 years. Eur Heart J. (2013) 34:2862–72. 10.1093/eurheartj/eht330 24086085

[22] D'Agostino RS Jacobs JP Badhwar V Paone G Rankin JS Han JM. The society of thoracic surgeons adult cardiac surgery database: 2017 update on outcomes and quality. Ann Thorac Surg. (2017) 103:18–24. 10.1016/j.athoracsur.2016.11.001 27884412

[23] Laffey JG Boylan JF Cheng DCH. The systemic inflammatory response to cardiac surgery. Anesthesiology (2002) 97:215–52. 10.1097/00000542-200207000-00030 12131125

[24] Kats S Brands R Hamad MAS Seinen W Scharnhorst V Wulkan RW. Prophylactic treatment with alkaline phosphatase in cardiac surgery induces endogenous alkaline phosphatase release. Int J Artif Organs (2012) 35:144–51. 10.5301/ijao.5000039 22395920

[25] Lauffenburger D Keller KH. Effects of leukocyte random motility and chemotaxis in tissue inflammatory response. J Theor Biol. (1979) 81:475–503. 10.1016/0022-5193(79)90047-X 395370

[26] Lauffenburger D Aris R Keller K. Effects of cell motility and chemotaxis on microbial population growth. Biophys J. (1982) 40:209–19. 10.1016/S0006-3495(82)84476-7 7183335 PMC1328997

[27] Lauffenburger DA Kennedy CR. Localized bacterial infection in a distributed model for tissue inflammation. J Math Biol. (1983) 16:141–63. 10.1007/BF00276054 6827185

[28] Reynolds A Rubin J Clermont G Day J Vodovotz Y Bard Ermentrout G. A reduced mathematical model of the acute inflammatory response: I. Derivation of model and analysis of anti-inflammation. J Theor Biol. (2006) 242:220–36. 10.1016/j.jtbi.2006.02.016 16584750

[29] Kumar R Clermont G Vodovotz Y Chow CC. The dynamics of acute inflammation. J Theor Biol. (2004) 230:145–55. 10.1016/j.jtbi.2004.04.044 15321710

[30] Dunster JL Byrne HM King JR. The resolution of inflammation: a mathematical model of neutrophil and macrophage interactions. Bull Math Biol. (2014) 76:1953–80. 10.1007/s11538-014-9987-x 25053556

[31] Su B Zhou W Dorman KS Jones DE Mathematical modelling of immune response in tissues. Comput Math Methods Med. (2009) 10:9–38. 10.1080/17486700801982713

[32] Pigozzo AB Macedo GC dos Santos RW Lobosco M. On the computational modeling of the innate immune system. BMC Bioinformatics (2013) 14(Suppl. 6):S7. 10.1186/1471-2105-14-S6-S7 23734602 PMC3633047

[33] Andrew SM Baker CTH Bocharov GA Rival approaches to mathematical modelling in immunology. J Comput Appl Math. (2007) 205:669–86. 10.1016/j.cam.2006.03.035

[34] Janeway CA Travers P Walport M Shlomchik M Immunobiology: The Immune System In Health and Disease. New York, NY: Garland Science (2001).

[35] Damas P Ledoux D Nys M Vrindts Y De Groote D Franchimont P. Cytokine serum level during severe sepsis in human IL-6 as a marker of severity. Ann Surg. (1992) 215:356–62. 10.1097/00000658-199204000-00009 1558416 PMC1242452

[36] Flögel U Willker W Leibfritz D. Determination of *de novo* synthesized amino acids in cellular proteins revisited by 13C NMR spectroscopy. NMR Biomed. (1997) 10:50–8. 10.1002/(SICI)1099-1492(199704)10:2<50::AID-NBM450>3.0.CO;2-1 9267861

[37] Yoshikawa M Yamauchi K Masago H. *De novo* messenger rna and protein synthesis are required for phytoalexin-mediated disease resistance in soybean hypocotyls. Plant Physiol. (1978) 61:314–7. 10.1104/PP.61.3.314 16660282 PMC1091857

[38] Pike AF Kramer NI Blaauboer BJ Seinen W Brands R. A novel hypothesis for an alkaline phosphatase “rescue” mechanism in the hepatic acute phase immune response. Biochim Biophys Acta (2013) 1832:2044–56. 10.1016/j.bbadis.2013.07.016 23899605

[39] van der Graaf PH Benson N Systems Pharmacology: bridging systems biology and Pharmacokinetics-Pharmacodynamics (PKPD) in drug discovery and development. Pharm Res. (2011) 28:1460–4. 10.1007/s11095-011-0467-9 21560018

[40] Helmlinger G Al-Huniti N Aksenov S Peskov K Hallow KM Chu L Drug-disease modeling in the pharmaceutical industry - where mechanistic systems pharmacology and statistical pharmacometrics meet. Eur J Pharm Sci. (2017) 109:S39–46. 10.1016/j.ejps.2017.05.028 28506868

[41] Gadkar K Kirouac DC Mager DE Van Der Graaf PH Ramanujan S. A six-stage workflow for robust application of systems pharmacology. CPT Pharmacometr Syst Pharmacol. (2016) 5:235–49. 10.1002/psp4.12071 27299936 PMC4879472

