# Supplementary material for: Supplemented Alkaline Phosphatase Supports the Immune Response in Patients Undergoing Cardiac Surgery: Clinical and Computational Evidence
Source: Front Immunol. 2018 Oct 11;9:2342. doi: 10.3389/fimmu.2018.02342 (PMC6193081; doi:10.3389/fimmu.2018.02342)
Supplement: Supplementary file 1 [file Data_Sheet_1.pdf]

Supporting Information for the paper

Presbitero A, Mancini E, Brands R, Krzhizhanovskaya VV and Sloot PMA (2018) Supplemented Alkaline Phosphatase Supports the Immune Response in Patients Undergoing Cardiac Surgery: Clinical and Computational Evidence. Front. Immunol. 9:2342. doi: 10.3389/fimmu.2018.02342

Table of Contents

1. Clinical Data 1
2. Model Terms and Biological Mechanisms 3
   2.1. Activation and Inhibition of Resting Macrophages 3
   2.2. Induction of Endothelial Permeability 3
   2.3. Interaction of cells with Inflammation Triggering Moieties 4
   2.4. Phagocytosis of Apoptotic and Necrotic Neutrophils 4
   2.5. Induction of Inflammation Triggering Moieties 5
   2.6. Natural Decay of Species 5
   2.7. Production of Pro-inflammatory and Anti-inflammatory Cytokines 5
   2.8. Necrosis and Cytokines Delay 6
   2.9. Release of Endogenous Alkaline Phosphatase from the Liver 7
   2.10. Administration of Bolus Alkaline Phosphatase 8
   2.11. Induction of Alkaline Phosphatase 8
3. The Human Innate Immune System Model (HIIS) 9
4. Parameter Estimation 14
5. Parameter Optimization 15
6. Parameter Identifiability Using Profile Likelihood 16
7. Difference between Experimental Data and Model Output 17
8. Sensitivity Analysis 19
9. Human innate immune system model without the induction mechanism of TNAP 21
10. Conversion of Concentrations 23
   10.1. Alkaline Phosphatase 23
   10.2. Cytokines 23
References 23

1. Clinical Data

Table 1. Demographics overview. Patients were subdivided into two branches: 1) Placebo branch, where patients were not given bolus Alkaline Phosphatase (AP), and 2) bIAP branch, where patients are injected with initial concentration of 1000 IU AP and given a continuous bolus of AP for a period of 8 hours right after surgery.

bIAP	mean	SD	min	median	max	L-95% CL	U-95% CL	n
Age (years)	70.0	8.8	52.0	70.0	85.0	66.0	76.0	27
Height (cm)	170.3	10.4	149.0	174.0	188.0	165.0	178.0	27
Body weight (kg)	77.1	15.9	53.0	76.0	113.0	68.0	82.0	27
BMI (kg/m²)	26.5	4.5	20.6	25.0	37.6	23.6	29.0	27

This research is financially supported by the Ministry of Education and Science of the Russian Federation, Agreement #14.575.21.0161 (26/09/2017), Unique Identification RFMEFI57517X0161.

Placebo	mean	SD	min	median	max	L-95% CL	U-95% CL	n
Age (years)	69.5	8.0	53.0	71.0	84.0	63.0	76.0	26
Height (cm)	168.3	12.4	148.0	171.5	188.0	156.0	178.0	26
Body weight (kg)	81.4	9.1	64.0	81.5	98.0	73.0	89.0	26
BMI (kg/m²)	28.9	3.7	24.2	28.1	39.4	26.1	31.1	25
**Total**	**mean**	**SD**	**min**	**median**	**max**	**L-95% CL**	**U-95% CL**	**n**
Age (years)	69.7	8.3	52	70.0	85	67.4	72.0	53
Height (cm)	169.3	11.4	148	173	188	166.1	172.4	53
Body weight (kg)	79.2	13.1	53	78.0	113	75.6	82.8	53
Age (years)	27.7	4.3	20.6	26.7	39.4	26.5	28.9	53

Table 2. Type and method of cardioplegia used based on treatment branches.

	bIAP		Placebo		Total	
	N	%	N	%	N	%
Blood	15	55.6	13	50.0	28	52.8
Cristalloid	10	37.0	13	50.0	23	43.4
Warm	12	44.4	12	46.2	24	45.3
Cold	13	48.1	14	53.8	27	50.9
Antegrade	21	77.8	19	73.1	40	75.5
Retrograde	1	3.7	5	19.2	6	11.3
Total	27	100.0	26	100.0	53	100.0

Patients were chosen based on the following inclusion criteria: 1) Male or non-pregnant, non-lactating female patients of any race in the ages of >18 years. (only women without non-childbearing potential). 2) Patients scheduled for coronary artery bypass surgery with CPB. 3) Patients had a EuroSCORE (Appendix I) of ≥2 and ≤6. 4) Patients had given written informed consent prior to participation in the trial.

Medical history of the patient population is summarized in Table 3. Note that one patient in the placebo group marked with cardiovascular diseases was excluded in the clinical trial.

Table 3. Medical History of Patient Population.

	**bIAP**					**Placebo**				
	no		yes		Total	no		yes		Total
	N	%	N	%	N	N	%	N	%	N
Cardiovascular diseases	-	-	27	100	27	1	3.8	25	96.2	26
Diabetes	24	88.9	3	11.1	27	21	80.8	5	19.2	26
Rheumatism	26	96.3	1	3.7	27	26	100	-	-	26
Alzheimer D.	27	100	-	-	27	26	100	-	-	26
Psoriasis	26	96.3	1	3.7	27	25	96.2	1	3.8	26
Liver dysfunction	26	96.3	1	3.7	27	26	100	-	-	26

Gastrointestinal disturbance	22	81.5	5	18.5	27	24	92.3	2	7.7	26
COPD / Asthma	21	77.8	6	22.2	27	26	100	-	-	26
Blood transfusion	27	100	-	-	27	25	100	-	-	25
Recurrent infectious diseases	26	96.3	1	3.7	27	25	96.2	1	3.8	26
Acute trauma	26	96.3	1	3.7	27	25	100	-	-	25
Other	10	37.0	17	63.0	27	14	56	11	44.0	25

2. Model Terms and Biological Mechanisms

2.1. Activation and Inhibition of Resting Macrophages

As deduced from the prevalence of complications following cardiothoracic surgery, *i.e.* in kidney function and brain temporary cognitive impairment, the mere presence of inflammation triggering moieties (ITMs) as by-products of simultaneous insults, both local or transported into the tissue, is enough to activate resting macrophages (M_R) at a rate of $\phi_{M_R|ITM}$. The activation of M_R is inhibited by the presence of anti-inflammatory cytokines (ACH) at a rate of θ_{ACH} and consequently the concentration of M_A is reduced accordingly. This is motivated by the biological mechanism where increasing concentration of apoptotic neutrophils cleared by activated macrophages induces a higher concentration of ACH. In other words, the higher ACH concentration is, the slower the activation process of resting macrophages becomes.

$$-\frac{\phi_{M_R|ITM} M_R ITM_{tissue}}{1+\theta_{ACH} ACH} \quad (1)$$

2.2. Induction of Endothelial Permeability

We model diapedesis and the movement of primed cellular entities from the blood into the tissue compartment by a permeability factor of the endothelial membrane, to account for the macroscopic spatial dynamics (movement of cells between blood and tissue) involved in a systemic inflammatory response.

Pro-inflammatory cytokines (CH) indirectly result in opening up the endothelial barrier. This allows the migration of monocytes and circulating primed neutrophils, as well as plasma resident molecules like albumin and alkaline phosphatase, from the bloodstream into the tissue. We model endothelial permeability through Equation (2), where ζ could either be M_R, $N_{\boldsymbol{R}}$, ITM, or AP. P_ζ^{max} and P_ζ^{min} are the maximum and minimum endothelial permeability respectively, CH designates the concentration of pro-inflammatory cytokines, while K_{eqCH} is the concentration of CH that gives 50% of the maximum effect on permeability. The term $f_{dillution}$ is simply a dilution factor that represents the diffusion of immune cells from the bloodstream (5 liters) into the tissue (75 liters, assuming an 80 kg person)

$$f_{dillution}\left(P_\zeta^{max} - P_\zeta^{min}\right)\frac{CH}{CH + K_{eqCH}} + P_\zeta^{min} \tag{2}$$

Equation (2) is modelled using a Hill type equation to make the permeability of the endothelial barrier non-linearly dependent on the CH concentrations. These permeability factors are limited by a multiplier that depends on the represented entity.

2.3. Interaction of cells with Inflammation Triggering Moieties

Equation (3) models the interaction of cells with ITMs by $M_A, N_A, G,$ and $AP,$ where the λ coefficients correspond to the rates at which the ITMs are being detoxified by dephosphorylation in the tissue.

$$ITM_{tissue}(\lambda_{ITM|M_A}M_A + \lambda_{ITM|N_A}N_A + \lambda_{ITM|AP_{Etissue}}AP_{Etissue} + \lambda_{ITM|AP_{Stissue}}AP_{Stissue}) \tag{3}$$

2.4. Phagocytosis of Apoptotic and Necrotic Neutrophils

Activated neutrophils either go into apoptosis or necrosis depending on the scale of insult in the body. M_A then proceeds to dispose of these neutrophils through phagocytosis. This process is modelled by Equation (4) where η represents either apoptotic or necrotic state of neutrophils, and $\lambda_{M_A|ND_\eta}$ is the rate of phagocytosis.

$$\lambda_{M_A|ND_\eta} M_A ND_\eta \tag{4}$$

2.5. Induction of Inflammation Triggering Moieties

Neutrophils undergoing necrosis cause all of their cellular contents, with endogenous-ITM characteristics, to spill out into the surrounding tissue, thereby triggering a vigorous pro-inflammatory response (1). We model the induction of ITMs through Equation (5) where α_{ND_N} is the rates of induction of ITM due to necrotic neutrophils (ND_N).

$$\alpha_{ND_N} ND_N \tag{5}$$

2.6. Natural Decay of Species

HIIS key players can disappear from the system due to natural species decay, modelled in Equation (6) where μ_ψ represents the decay rate of species ψ.

$$-\mu_\psi \psi \tag{6}$$

2.7. Production of Pro-inflammatory and Anti-inflammatory Cytokines

Equation (7) models the production of CH, such as interleukin (IL)-1, and tumor necrosis factor (TNF), where the term $\beta_{M_A|ITM} M_A ITM_{tissue} + \beta_{N_A|ITM} N_A ITM_{tissue}$ describes the production of CH

when M_A and N_A phagocytose ITM. This mechanism is regulated by ACH. The larger the ACH concentration is, the slower the increase in CH. The term $1 - CH/CH^{max}$ models the growth factor of CH. Hence, CH is no longer produced when it reaches a maximum concentration of CH^{max} in the tissue.

$$\frac{(\beta_{M_A|ITM} M_A ITM_{tissue} + \beta_{N_A|ITM} N_A ITM_{tissue})(1 - \frac{CH}{CH^{max}})}{1 + \theta_{ACH} ACH} \tag{7}$$

ACH, such as $IL4$, $IL10$, and IFN-alpha, are actively produced by M_A, as well as when M_A phagocytoses ND_A. This is modelled by the term $\left(\beta_{ND_A|M_A} M_A ND_A + \alpha_{ACH|M_A} M_A\right)$ in Equation (8). The production of ACH is limited by the maximum concentration of ACH allowed in the tissue (ACH^{max}) through a growth factor represented by the term $1 - ACH/ACH^{max}$.

$$(\beta_{ND_A|M_A} M_A ND_A + \alpha_{ACH|M_A} M_A)(1 - \frac{ACH}{ACH^{max}}) \tag{8}$$

2.8. Necrosis and Cytokines Delay

Neutrophils naturally die through a programmed death process called "apoptosis." However, if the stimulus is too strong, neutrophils go into the necrotic state, which is a turbulent cell death that triggers the release of tremendous amounts of ITMs in the surrounding tissue. It remains unclear how much of this activated neutrophil concentration goes into apoptosis, or conversely into necrosis. We simplify these mechanisms in our model by assuming that necrosis takes place when activated neutrophils phagocytose ITMs. Therefore, the larger the insult, the more ITMs there are in the body and the higher the concentration of necrotic neutrophils becomes. Apoptosis, on the other hand is simply modelled by the decay of activated neutrophils and is inversely proportional to the

concentration of ITMs in the system. These mechanisms are modelled by Equation (9), where the term on the left corresponds to apoptosis, and the term on the right to necrosis with temporal delay $t_{NDNdelay}$ of 5 hours.

$$-\mu_{ND_A} \frac{N_A}{1 + ITM_{tissue}} - \frac{1}{1 + exp^{-r_{\mathrm{NDN}}(t - t_{NDNdelay})}} \lambda_{ITM|N_A} N_A ITM_{tissue} \tag{9}$$

It is also of great importance to model the delay of the cytokine response. This was observed in patients by cytokines increment in the plasma after about 20 minutes. We model this delay mechanism as a step function given by Equation (10) where b is the rate which we set to maximum to account for the immediate increase in cytokine population as soon as the time reaches $t_{CHdelay}$.

$$\frac{1}{1 + exp^{-b(t - t_{CHdelay})}} \tag{10}$$

2.9. Release of Endogenous Alkaline Phosphatase from the Liver

Alkaline phosphatase in the bloodstream is used up in numerous ways. The body then establishes homeostasis by releasing a considerable amount of liver-derived AP (about 50% of total plasma concentration of AP) into the blood circulation through a process described by Pike et al., (2013). Note that the largest amount of AP in the body is stored at the bile canalicular membrane. We model this supply of AP through a Gaussian function that is centred around zero $AP_{Eblood} + AP_{Sblood}$ concentration, which served as the body's "distress signal" to fire up the liver into releasing its contents (indirectly) into the blood stream. 'w' is the width of the Gaussian function that sets the AP "distress signal" concentration and $r_{distress}$ is the rate at which AP is released into circulation by the liver.

$$r_{distress}\left(exp^{-\frac{(AP_{Eblood}+AP_{Sblood})^2}{2w^2}}\right) \quad (11)$$

2.10. Administration of Bolus Alkaline Phosphatase

In APPIRED II, a total amount of 10,000 units AP (1000 IU bolus and 9000 IU infusion) was administered over a period of 8 hours. AP infusion is modelled by a step function given by Equation (12) where b is set to maximum to represent the sudden decline in bIAP concentration as soon as the infusion has been terminated at t_{stop}.

$$r_{inject}\left(\frac{1}{1+exp^{b(t-t_{suppstop})}}\right) \quad (12)$$

2.11. Induction of Alkaline Phosphatase

The intricate details pertaining to the underlying mechanisms to how alkaline phosphatase is induced upon a systemic insult are still unknown and not found anywhere in literature. We propose a hypothesis that the induced AP is only proportional to the amount of supplemented AP that is in the system. We model this induction mechanism as the inverse of a sigmoid function where the amplitude is dependent on parameter $r_{inducepeak}$ and the total concentration of supplemented AP that is in the system $(AP_{Stissue} + AP_{Sblood})$. Note that the induction mechanism has a delay of $t_{APdelay}$, which refers to the time it takes for the liver to be able to supply AP again, having emptied out 1000 IU at the onset of surgery.

$$\frac{r_{inducepeak}}{1+exp^{r_{induce}(t-t_{APdelay})}}\,(AP_{Stissue} + AP_{Sblood}) \quad (13)$$

3. The Human Innate Immune System Model (HIIS)

The HIIS model is summarized in equations (14)-(27). Equations (14)-(19) model the biological mechanisms in the blood compartment while equations (20)-(27) correspond to the tissue compartment. The parameters and initial values that we used in the model are those from literature, as well as those indicated in the cardiac surgery clinical study.

Blood Compartment

$$\frac{dITM_{blood}}{dt} = -\mu_{ITM} ITM_{blood} - ITM_{blood}\left(\lambda_{ITM|AP_E} AP_{Eblood} + \lambda_{\mathrm{ITM}|AP_S} AP_{Stissue}\right) - \left[\left(P_{ITM}^{max} - P_{ITM}^{min}\right)\frac{CH}{CH + K_{eqCH}} + P_{ITM}^{min}\right] ITM_{blood}\left(1 - \frac{ITM_{tissue}}{ITM_{max}}\right) + \frac{r_{ITMpeak}}{1 + exp^{r_{ITM}(t - t_{ITM})}}\left(1 - \frac{ITM_{blood}}{ITM_{max}}\right) \tag{14}$$

$$\frac{dAP_{Eblood}}{dt} = -\mu_{AP_E} AP_{Eblood} - [(P_{AP}^{max} - P_{AP}^{min})\frac{CH}{CH + K_{eqCH}} + P_{AP}^{min}\ ] AP_{Eblood} - \lambda_{ITM|AP_E} ITM_{blood} AP_{Eblood} + \frac{1}{1 + exp^{-r_{AP}(t - t_{APdelay})}}\left[r_{distress}\left(exp^{-\frac{(AP_{Eblood} + AP_{Sblood}\ )^2}{2w^2}}\right) + \frac{r_{inducepeak}}{1 + exp^{r_{induce}(t - t_{APdelay})}}\ (AP_{Stissue} + AP_{Sblood})\right] \tag{15}$$

$$\frac{dAP_{Sblood}}{dt} = -\mu_{AP_S} AP_{Sblood} - f_{dillution}.[(P_{AP}^{max} - P_{AP}^{min})\frac{CH}{CH + K_{eqCH}} + P_{AP_E}^{min}\ ] AP_{Sblood} + r_{inject}(\frac{1}{1 + exp^{b(t - t_{suppstop})}}) - \lambda_{ITM|AP_S} ITM_{blood} AP_{Sblood} \tag{16}$$

$$\frac{dN_R}{dt} = -\mu_{N_R} N_R - \left[P_{N_R}^{max}\frac{CH}{CH + K_{eqCH}}\right]\left(1 - \frac{N_A}{N_R^{max}}\right) N_R + r_{Nhomeo}(1 - \frac{N_R}{N_R^{max}}) N_R \tag{17}$$

$$\frac{dCH}{dt} = -\mu_{CH} CH + \frac{1}{1 + exp^{-b(t - t_{CHdelay})}} [\frac{(\beta_{M_A|ITM} M_A ITM_{tissue} + \beta_{N_A|ITM} N_A ITM_{tissue})(1 - \frac{CH}{CH^{max}})}{1 + \theta_{ACH} ACH}] \tag{18}$$

$$\frac{dACH}{dt} = -\mu_{ACH} ACH + (\beta_{ND_A|M_A} M_A ND_A + \alpha_{ACH|M_A} M_A)(1 - \frac{ACH}{ACH^{max}}) \tag{19}$$

Tissue Compartment

$$\frac{dITM_{tissue}}{dt} = -\mu_{ITM}ITM_{tissue} - ITM_{tissue}(\lambda_{ITM|M_A}M_A + \lambda_{ITM|N_A}N_A + \lambda_{ITM|AP_{Etissue}}AP_{Etissue} + \lambda_{ITM|AP_{Stissue}}AP_{Stissue}) + \alpha_{ND_N}ND_N + f_{dillution}[(P_{ITM}^{max} - P_{ITM}^{min})\frac{CH}{CH + K_{eqCH}} + P_{ITM}^{min}]ITM_{blood}(1 - \frac{ITM_{tissue}}{ITM_{max}}) \quad (20)$$

$$\frac{dM_R}{dt} = -\mu_{M_R}M_R - \frac{\phi_{M_R|ITM}M_R ITM_{tissue}}{1 + \theta_{ACH}ACH} + f_{dillution}[(P_{M_R}^{max} - P_{M_R}^{min})\frac{CH}{CH + K_{eqCH}} + P_{M_R}^{min}]\ [M^{max} - (M_R + M_A)] \quad (21)$$

$$\frac{dM_A}{dt} = -\mu_{M_A}M_A + \frac{\phi_{M_R|ITM}M_R ITM_{tissue}}{1 + \theta_{ACH}ACH} \quad (22)$$

$$\frac{dN_A}{dt} = -\mu_{ND_A}\frac{N_A}{1 + ITM_{tissue}} - \frac{1}{1 + exp^{-r_{\text{NDN}}(t - t_{NDNdelay})}}\lambda_{ITM|N_A}N_A ITM_{tissue} + f_{dillution}[P_{N_R}^{max}\frac{CH}{CH + K_{eqCH}}](1 - \frac{N_A}{N_R^{max}})N_R \quad (23)$$

$$\frac{dND_A}{dt} = \mu_{ND_A}\frac{N_A}{1 + ITM_{tissue}} - \lambda_{ND_A|M_A}M_A ND_A \quad (24)$$

$$\frac{dND_N}{dt} = \frac{1}{1 + exp^{-r_{\text{NDN}}(t - t_{NDNdelay})}}\lambda_{ITM|N_A}N_A ITM_{tissue} - \lambda_{ND_N|M_A}M_A ND_N \quad (25)$$

$$\frac{dAP_{Etissue}}{dt} = -\mu_{AP_E}AP_{Etissue} + f_{dillution}[(P_{AP}^{max} - P_{AP}^{min})\frac{CH}{CH + K_{eqCH}} + P_{AP}^{min}]AP_{Eblood} - \lambda_{ITM|APE}ITM_{tissue}AP_{Etissue} \quad (26)$$

$$\frac{dAP_{Stissue}}{dt} = -\mu_{AP_S}AP_{Stissue} + f_{dillution}[(P_{AP}^{max} - P_{AP}^{min})\frac{CH}{CH + K_{eqCH}} + P_{AP_E}^{min}]AP_{Sblood} - \lambda_{ITM|APS}ITM_{tissue}AP_{Stissue} - \lambda_{ITM|APS}ITM_{tissue}AP_{Stissue} \quad (27)$$

Table 4. Summary of Terms and Description Used in the Human Innate Immune System Model. Note that the values indicated for Immune cells (entries 1-14) are initial values.

	Category	Equation Terms	Description	Value	Unit	Reference
1	Immune Cell	ITM_{blood}	concentration of Inflammation Triggering Moeties (ITMs) in the bloodstream	$3.5x10^7$	$\frac{molecules}{mm^3}$	(2)
2		AP_{Eblood}	concentration of endogenous Alkaline Phosphatase (AP) in the bloodstream	4000	IU	(3)

3		AP_{Sblood}	concentration of supplemented AP in the bloodstream	1000	IU	experiment*
4		N_R	concentration of resting neutrophils	$1.2x10^5$	$\frac{cells}{mm^3}$	(4)
5		CH	concentration of pro-inflammatory cytokines	0	$\frac{molecules}{mm^3}$	(5)
6		ACH	concentration of anti-inflammatory cytokines	0	$\frac{molecules}{mm^3}$	(5)
7		ITM_{tissue}	concentration of ITMs in the tissue	$3.5x10^5$	$\frac{molecules}{mm^3}$	(5)
8		M_R	concentration of resting macrophages	$1.5x10^3$	$\frac{cells}{mm^3}$	(4)
9		M_A	concentration of activated macrophages	0	$\frac{cells}{mm^3}$	(5)
10		N_A	concentration of activated neutrophils	0	$\frac{cells}{mm^3}$	(5)
11		ND_A	concentration of apoptotic neutrophils	0	$\frac{cells}{mm^3}$	(5)
12		ND_N	concentration of necrotic neutrophils	0	$\frac{cells}{mm^3}$	(5)
13		$AP_{Etissue}$	concentration of endogenous AP in the tissue	2500	IU	experiment*
14		$AP_{Stissue}$	concentration of supplemented AP in the tissue	0	IU	experiment*
15	Rate of Decay	μ_{ITM}	Decay rate of ITMs	$4.4x10^{-6}$	$1/min$	(4)
16		μ_{AP_E}	Decay rate of endogenous AP	$6.3x10^{-4}$	$1/min$	(6)
17		μ_{M_R}	Decay rate of resting macrophages	$2.9x10^{-6}$	$1/min$	(4)
18		μ_{ND_A}	Decay rate of necrotic neutrophils	-	-	calibrated (see Table 5)
20		μ_{AP_S}	Decay rate of supplemented AP	0.1	$1/min$	experiment*
21	Rate of Phagocytosis of Inflammation Triggering Moieties	$\lambda_{ITM\|AP_E}$	neutralization rate of ITMs by endogenous AP	$3.9x10^{-9}$	$\frac{mm^3}{molecules.min}$	(7)
22		$\lambda_{ITM\|AP_S}$	neutralization rate of ITMs by supplemented AP	$2.6x10^{-9}$	$\frac{mm^3}{molecules.min}$	(7)
23		$\lambda_{ITM\|M_A}$	phagocytosis rate of ITMs by activated macrophages	$7x10^{-4}$	$\frac{mm^3}{cells.min}$	(4)
24		$\lambda_{ITM\|N_A}$	phagocytosis rate of ITMs by activated neutrophils	-	-	calibrated (see Table 5)
25		$\lambda_{ITM\|AP_{Etissu}}$	phagocytosis rate of ITMs by endogenous AP in tissue	$3.9x10^{-9}$	$\frac{mm^3}{molecules.min}$	(7)
26		$\lambda_{ITM\|AP_{Stissu}}$	phagocytosis rate of ITMs by supplemented AP in tissue	$2.6x10^{-9}$	$\frac{mm^3}{molecules.min}$	(7)
27	Rate of Engulfment of Neutrophils	$\lambda_{ND_A\|M_A}$	rate at which necrotic neutrophils are engulfed by macrophages	-	-	calibrated (see Table 5)
28		$\lambda_{ND_A\|M_A}$	rate at which apoptotic neutrophils are engulfed by macrophages	-	-	calibrated (see Table 5)
29	Permeability Constants	K_{eqCH}	concentration of pro-inflammatory cytokines that give 50% of the maximum effect of permeability	-	-	calibrated (see Table 5)

30		P_{AP}^{max}	maximum permeability of AP	-	-	calibrated (see Table 5)
31		P_{AP}^{min}	minimum permeability of AP	-	-	calibrated (see Table 5)
32		$P_{N_R}^{max}$	maximum permeability of resting neutrophils	-	-	calibrated (see Table 5)
33	Maximum Concentration of Immune Cells	ITM_{max}	maximum concentration of ITMs	$3.6x10^7$	$\frac{molecules}{mm^3}$	(8)
34		N_R^{max}	maximum concentration of resting neutrophils	$2.5x10^3$	$\frac{cells}{mm^3}$	(5)
35		ACH^{max}	maximum concentration of anti-inflammatory cytokines	$5.8x10^6$	$\frac{molecules}{mm^3}$	(9)
36		M^{max}	maximum concentration of Monocytes	$1.5x10^2$	$\frac{cells}{mm^3}$	(5)
37	Time Delay	t_{ITM}	delay in generating ITMs at the onset of surgery	10	min	correspondence with Ruud Brands
38		$t_{APdelay}$	delay at which AP is supplied into the bloodstream	60	min	correspondence with Ruud Brands
39		$t_{suppstop}$	time at which bolus AP that is injected into the bloodstream is stopped	480	min	experiment*
40		$t_{CHdelay}$	delay of cytokine recruitment	20	min	correspondence with Ruud Brands
41		$t_{NDNdelay}$	delay of necrosis	300	min	correspondence with Ruud Brands
42	Rate of Increase/Decrease of Sigmoid Function	r_{ITM}	rate of increase of ITMs at the onset of surgery	-	-	calibrated (see Table 5)
43		r_{AP}	rate of increase at which AP is supplied into the bloodstream	-	-	calibrated (see Table 5)
44		r_{induce}	rate of induction of AP in the supplemented branch, where patients are supplied initially with 1000 IU of AP, and continuously with bolus AP	-	-	calibrated (see Table 5)
45		r_{NDN}	rate at which activated neutrophils become necrotic	-	-	calibrated (see Table 5)
46		b	set to maximum to signify an immediate rise/fall of concentration	-	-	-
47	Maximum Peak Saturation of Sigmoid Function	$r_{ITMpeak}$	peak concentration of ITMs	-	-	calibrated (see Table 5)
48		$r_{distress}$	peak concentration of AP that is supplied into the bloodstream to establish homeostasis	-	-	calibrated (see Table 5)
49		$r_{inducepeak}$	peak concentration of induced AP	-	-	calibrated, (see Table 5)
50		r_{inject}	peak concentration of injected bolus AP	-	-	experiment*
51	Pro-inflammatory Cytokine	$\beta_{M_A\|ITM}$	rate of pro-inflammatory cytokine production when activated macrophages	-	-	calibrated (see Table 5)

	Production		phagocytose ITMs			
52		$\beta_{N_A\|ITM}$	rate of pro-inflammatory cytokine production when activated neutrophils neutralize ITMs	-	-	calibrated (see Table 5)
53		θ_{ACH}	rate at which anti-inflammatory cytokines inhibit pro-inflammatory cytokine production	-	-	calibrated (see Table 5)
54	Anti-inflammatory Cytokine Production	$\beta_{ND_A\|M_A}$	rate at which anti-inflammatory cytokines are produced when activated macrophages engulf apoptotic neutrophils	-	-	calibrated (see Table 5)
55		$\alpha_{ACH\|M_A}$	rate at which anti-inflammatory cytokines are produced by activated macrophages	$1.3x10^{-3}$	$1/min$	(5)
56	Dilution Factor	$f_{dillution}$	dilution factor that accounts for the movement of immune cells from the bloodstream into the tissue	$1/16$		(10)
57	Positive Feedback	α_{ND_N}	rate of production of ITMs by necrotic neutrophils	-	-	calibrated (see Table 5)
58		$\phi_{M_R\|ITM}$	rate at which resting macrophages are activated by ITMs	$7.3x10^{-5}$	$\frac{mm^3}{molecules.min}$	(5)
59		r_{Nhomeo}	rate of homeostasis of resting neutrophils in the bloodstream	-	-	calibrated (see Table 5)
60	Homeostasis of Neutrophils	w	defines how long the concentration of neutrophils are replenished back into the bloodstream to establish homeostasis	-	-	calibrated (see Table 5)

*concentration used in the clinical study

4. Parameter Estimation

A cost function quantifies how closely the output of the model agrees with the experimental data using a set of parameters θ. This cost function is normally designated by the root mean of square error as shown in Equation (28):

$$\chi^2(\theta) = \sum_{k=1}^{m} \sqrt{\frac{\sum_{l=1}^{d}\left(\widetilde{y_{kl}^{D}} - \widetilde{y_k(\theta, t_l)}\right)^2}{d_k}} \tag{28}$$

where y_{kl}^{D} corresponds to the d data points for each observable k (Alkaline Phosphatase, anti- and pro-inflammatory cytokines) at time points t_l. d_k is the number of data points for observable k, and $y_k(\theta, t_l)$ is the model output for observable k at time t_l using the parameters θ. We also normalized the experimental data and the model output for observable k by dividing them with the maximum experimental value and model output value respectively. In this way, we assign equal contributions from each dataset to the objective function especially since they have different range of values.

$$\widetilde{y_{kl}^{D}} = \frac{y_{kl}^{D}}{max(y_{kl}^{D})}, \widetilde{y_k(\theta, t_l)} = \frac{y_k(\theta, t_l)}{max(y_{kl}^{D})} \tag{29}$$

The set of parameters θ can be estimated numerically by minimizing $\chi^2(\theta)$:

$$\hat{\theta} = argmin[\chi^2(\theta)] \tag{30}$$

5. Parameter Optimization

We estimate model parameters by minimizing the cost function in Equation (30). Here we used the truncated Newton method, also known as Newton-Conjugate Gradient method, which solves large nonlinear optimization problems given a set constraints or boundaries for each parameter in θ.

The NCG method is an iterative algorithm that searches for optimal parameters corresponding to the minimal cost function in the direction of p_k^N as summarized in Equation (31)

$$p_k^N = -\nabla^2 f_k^{-1} \nabla f_k \tag{31}$$

where p_k^N is the search direction, $\nabla^2 f_k$ is the the second derivative of the cost function (Hessian matrix), and ∇f_k is the gradient of the cost function at an optimization step k (11). However, the Hessian matrix $\nabla^2 f_k$ may not always be positive definite and the step direction p_k^N may not always be in the descending direction. The NCG method avoids this issue using the conjugate gradient method, which terminates the iteration steps if a negative curvature of the Hessian matrix is encountered. Additionally, the NCG method is a Hessian-free method because it does not require explicit knowledge of the Hessian matrix. Rather, it only needs to calculate for the matrix-vector products between the Hessian matrix $\nabla^2 f_k$ and the search vector p, which can be solved via the finite-difference technique. A more detailed discussion of the conjugate gradient method and the finite-difference techniques can be found in (11).

Table 5. Summary of calibrated model parameters. Note that the calibration range excludes 0.

	Model Parameters	Unit	Calibrated Supplemented AP Experiment	Calibration Range	Reference
1	$\beta_{M_A\|ITM}$	$\frac{mm^3}{cells.min}$	7.8×10^{-4}	(0,1]	constrained (8)
2	$\beta_{N_A\|ITM}$	$\frac{mm^3}{cells.min}$	4.8×10^{-2}	(0,1]	constrained (8)
3	θ_{ACH}	$\frac{mm^3}{molecules}$	1×10^{-10}	(0,1]	constrained (8)
4	$\beta_{ND_A\|M_A}$	$\frac{molecules.mm^3}{cells^2.min}$	9×10^1	$(0, 10^4]$	constrained (12)
5	$\lambda_{ITM\|N_A}$	$\frac{mm^3}{cells.min}$	1×10^{-6}	(0,10]	constrained (12)
6	$\alpha_{ND_N,}$	$\frac{molecules}{cells.min}$	1×10^3	$[10^2, 10^4]$	constrained (1)
7	P_{AP}^{max}	$1/min$	2×10^{-3}	(0,1]	constrained (data)
8	P_{AP}^{min}	$1/min$	2×10^{-4}	(0,0.1]	constrained (data)
9	$r_{distress}$	$\frac{molecules}{mm^3.min}$	3×10^6	$[10^5, 10^9]$	constrained (13)
10	w	$\frac{molecules}{mm^3}$	8×10^7	$[10^5, 10^9]$	constrained (13)
11	$r_{inducepeak}$	$1/min$	21	$(0, 100]$	constrained (data)
12	r_{induce}	$1/min$	5×10^{-2}	(0,10]	constrained (data)
13	r_{AP}	$1/min$	8×10^{-2}	(0,1]	constrained (data)
14	r_{ITM}	$1/min$	5×10^{-1}	(0,1]	constrained (8)
15	$r_{ITMpeak}$	$\frac{molecules}{mm^3.min}$	5×10^{12}	$[10^{13}, 10^{14}]$	constrained (8)
16	r_{NDN}	$1/min$	8×10^{-3}	(0,1]	constrained (13)
17	$\lambda_{ND_N\|M_A}$	$\frac{mm^3}{cells.min}$	5×10^{-6}	(0,1]	constrained (13)
18	$\lambda_{ND_A\|M_A}$	$\frac{mm^3}{cells.min}$	3×10^{-5}	(0,1]	constrained (13)
19	μ_{ND_A}	$1/min$	2.5×10^1	$[10^1, 10^4]$	constrained (13)
20	K_{eqCH}	$\frac{molecules}{mm^3}$	2×10^4	$[10^4, 10^7]$	constrained (13)
21	r_{homeo}	$1/min$	1×10^{-4}	(0,1]	constrained (13)
22	$P_{N_R}^{max}$	$1/min$	6×10^{-3}	(0,1]	constrained (13)

6. Parameter Identifiability Using Profile Likelihood

Although we propose a model of the human innate immune response that aims at describing the dynamics of fourteen (14) immune cells and molecules that are located either in the bloodstream or in the tissue, clinical data is only available for AP, pro-inflammatory, and anti-inflammatory cytokines. Given the noise in the experimental data, the assurance of model parameters being estimated

unambiguously becomes challenging. More often than not, experimental data is insufficient especially with respect to the size of the model, hence estimated parameters can be non-identifiable (14).

Parameter identifiability analysis addresses a crucial step in estimating parameters. This type of analysis tries to answer whether it is possible to recover a unique set of model parameters given a dataset. One tool that was developed to evaluate which parameters are identifiable, and hence consequently infer the feasibility of the model, was introduced by Rau et al in (15) through a technique called *profile likelihood*. This type of approach aims at "profiling" a single parameter by fixing its value across a range of values, while fitting the remaining parameters using the objective function in Equation (28). The minimum value of the objective function constitutes the likelihood profile for the fixed parameter within the computed confidence interval derived using a threshold in the likelihood. Due to the intense computational requirements of a detailed parameter identifiability study, we performed a preliminary study (data not shown) focusing on the sensitive parameters identified in the global sensitivity analysis (see Section 8). This preliminary study shows that all sensitive parameters are either identifiable or practically non-identifiable parameters. Additionally for all practically non-identifiable parameters the issue is related to the lower boundary, which cannot be lower than 0, while the upper boundary always crosses the threshold in the likelihood.

7. Difference between Experimental Data and Model Output

We show the normalized residuals between experimental data and results of the model simulation for Alkaline Phosphatase, pro-inflammatory, and anti-inflammatory cytokines by calculating the difference between experimental data and model output divided by the experimental data. Normalized residual values are at maximum between 0-5 hours after surgery time for cytokines (see A-B). Considering the large standard deviation observed within the 0-5 hours post-surgery timeframe

on the cytokines dataset that in fact spans 7 orders of magnitude in units cells/m^3, our results still fall within this expected range. Note that patients' blood parameters usually stabilize to normal concentrations within this time period after surgery.

Normalized residuals for AP are only at maximum at the onset of surgery. This is as expected because right at the start of surgery, patients are injected with bovine AP concentration of 1000 IU. Bovine AP has a short half life and could disappear within 20 minutes. However, the earliest recorded AP concentration from the experimental data is 30 minutes after the start of operation. Therefore, we do not see a trace of this added bovine AP on the data, giving a residual that is close to the initial concentration of AP given to the patient.

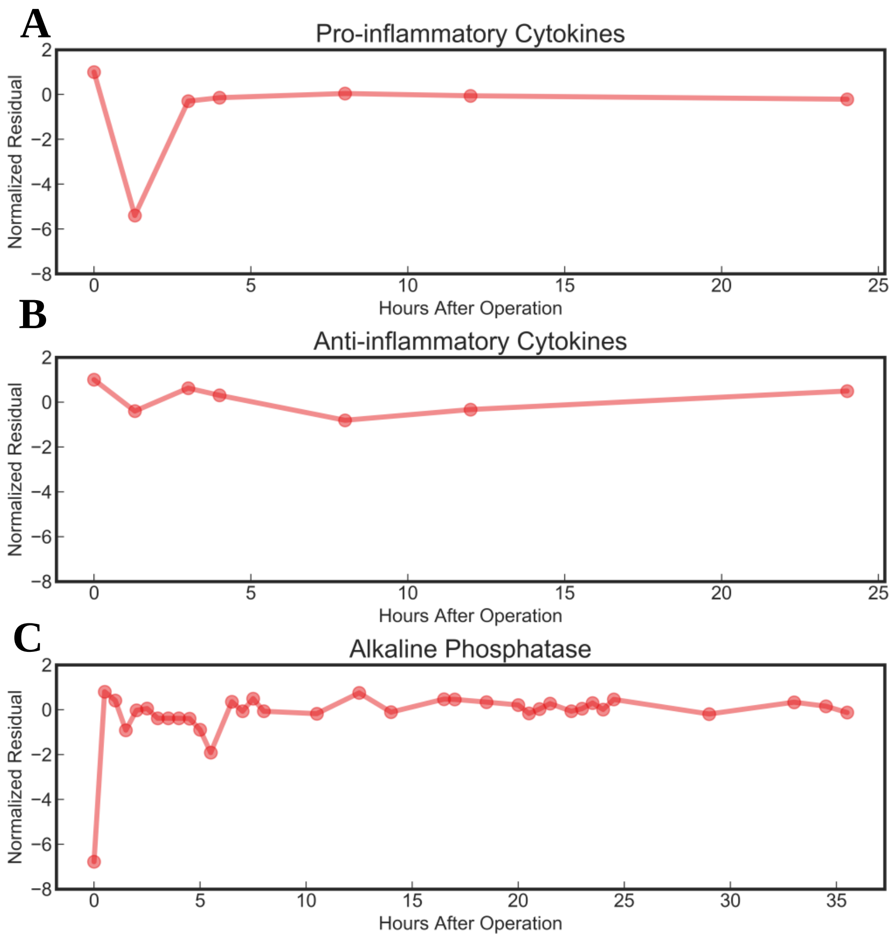

Figure 1. Normalized Residuals (Experimental Data– Predicted Value/Experimental Data) for A) Pro-inflammatory Cytokines, B) Anti-inflammatory Cytokines, and C) Alkaline Phosphatase.

8. Sensitivity Analysis

In order to obtain a deeper understanding of the model dynamics, it is important to quantify how the variability of model parameters affects the predictions of the model. For this reason, we perform a global sensitivity analysis (16) for three of the most significant model outputs: Alkaline Phosphatase, pro-inflammatory cytokines, and anti-inflammatory cytokines for which we have the experimental data. The strength of variance-based measures of sensitivity lies on the fact that they measure sensitivity across the entire input space and could deal with nonlinear responses and measure the effect of interactions in non-additive systems. We chose the Fourier amplitude sensitivity test (FAST) due to its computational efficiency (17–20).

The ranges of parameter values were varied in a range of biologically justified values given in Table 5. The 22 parameters of the model were then sampled using a periodic sampling approach with the number of parameter samples N = 5000 to ensure an adequate coverage of the multidimensional parameter space. We analyzed the sensitivity of the model by plotting the sensitivity indices with 95% confidence of the FAST method (see Figure 2).

We show that concentrations of pro-inflammatory cytokines are most sensitive to θ_{ACH}, $\beta_{M_A|ITM}$, $\beta_{ND_A|M_A}$, and r_{NDN} (see Figure 2A) in various time intervals. r_{NDN} only becomes sensitive 30 minutes after the onset of surgery. This is due to the delay in the recruitment of neutrophils into the tissue. The sensitivity indices for $\beta_{M_A|ITM}$, θ_{ACH}, on the other hand peak right after the onset of surgery and decrease beyond this time interval. These parameters are sensitive because they are incorporated in the model Equation (18) which regulates the concentration of pro-inflammatory cytokines. The interactions between activated neutrophils and ITMs also trigger the recruitment of pro-inflammatory cytokines. Hence, the term r_{NDN}, which controls the activation rate of resting neutrophils in Equation (23), turns out to be sensitive as well.

$\beta_{ND_A|M_A}$, $\beta_{M_A|ITM}$, θ_{ACH}, $\lambda_{ND_N|M_A}$, K_{eqCH}, and $P_{N_R}^{max}$ are the most sensitive parameters among those investigated with respect to the concentration of anti-inflammatory cytokines as shown in Figure 10B. The first three parameters are the same for both pro-inflammatory cytokines and anti-inflammatory cytokines because these two immune cells depend on each other. However, for anti-inflammatory cytokines the sensitivity is more pronounced as the anti-inflammatory response continues for several hours after surgery.

Finally, the parameters shown to be most sensitive for the production of endogenous AP in the bloodstream are w, r_{AP}, $r_{distress}$ and P_{AP}^{min}. These parameters have direct effect on the concentration of endogenous AP as can be seen in Equation (15) of the model. The sensitivity of P_{AP}^{min} to endogenous AP shows an initial peak within the first hour, then it goes down and rises 40 minutes after surgery. Note that bolus AP is continuously injected up until 8 hours after surgery. Therefore, the effect of adjusting the minimum permeability of AP (P_{AP}^{min}) that allows the transfer from the bloodstream into the tissue is also minimal within this time period.

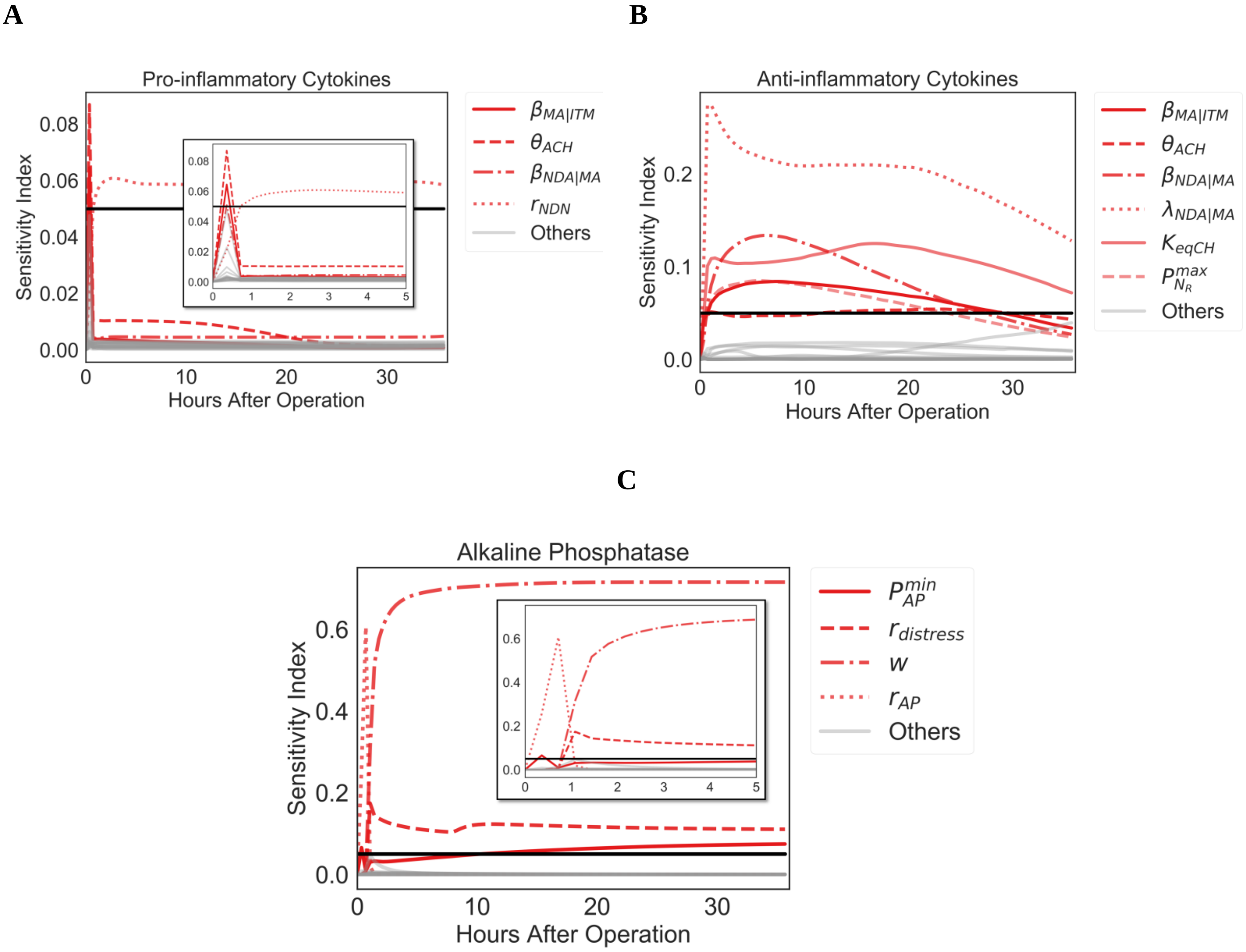

Figure 2. Fourier Amplitude Sensitivity Analysis (FAST) sensitivity indices of model parameters with respect to A) Pro-inflammatory Cytokines, B) Anti-inflammatory Cytokines, and C) Alkaline Phosphatase Concentrations up until 36 hours after surgery. The red lines correspond to parameters that are the most sensitive while the gray lines are those that are not. Note that the black horizontal line determines the confidence level of 95%. The closer the index is to 1, the more sensitive the model prediction is to a parameter.

9. Human innate immune system model without the induction mechanism of TNAP

HIIS model without the induction term predicts exactly the same dynamics for ITMs in plasma, ITMs in tissue and neutrophils in both branches as shown in Figure 3. Hence, without the added induced AP, immune cells in the supplemented branch exhibit the same dynamics as in placebo branch.

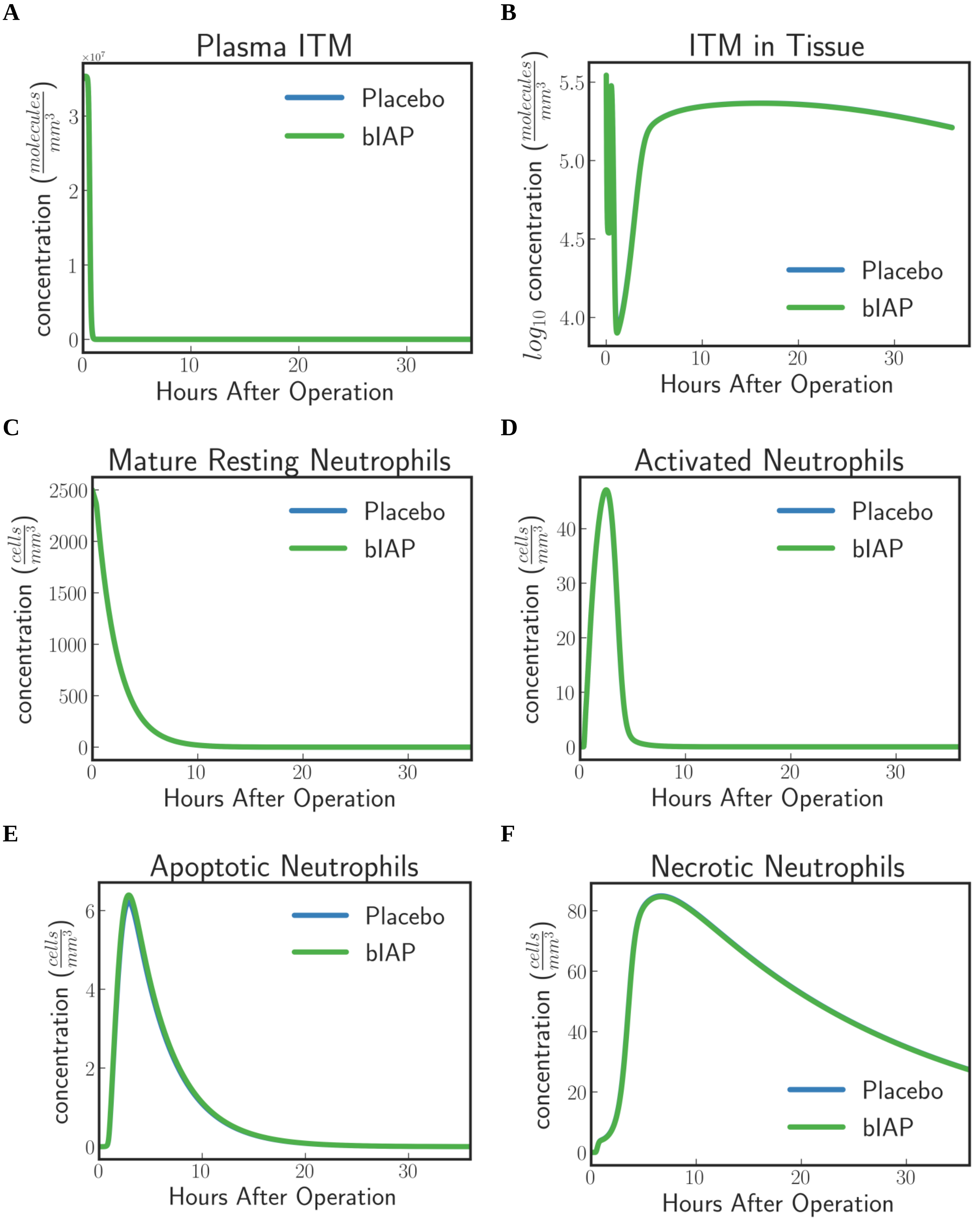

Figure 3. Dynamics of (**A**) ITMs in Plasma, (**B**) ITMs in Tissue, (**C**) Resting Neutrophils, (**D**) Activated Neutrophils, (**E**) Apoptotic Neutrophils and (**F**) Necrotic Neutrophils in the tissue. Dynamics of immune cells are similar for supplemented bIAP and placebo branches.

10. Conversion of Concentrations

10.1. Alkaline Phosphatase

$$AP\left(\frac{IU}{L}\right) = \mathrm{AP}\left(\frac{molecules}{mm^3}\right) \times \frac{1\,mole}{6.02 \times 10^{23}\,molecules} \times \frac{Molecular\ Weight_{AP}\ (g)}{1\,mole} \times \frac{Activity_{AP}\ (IU)}{1 \times 10^{-3} g} \times \frac{1 \times 10^6 mm^3}{1\,L} \tag{32}$$

where $Molecular\ Weight_{AP} = 160\ kg/mole$

10.2. Cytokines

$$cytokine\left(\frac{pg}{ml}\right) = cytokine\left(\frac{molecules}{mm^3}\right) \times \frac{1\,mole}{6.02 \times 10^{23}\,molecules} \times \frac{Molecular\ Weight_{cytokine}\ (g)}{1\,mole} \times \frac{1 \times 10^{12} pg}{1\,g} \times \frac{1 \times 10^6 mm^3}{1L} \times \frac{1L}{1 \times 10^3\,ml} \tag{33}$$

where $Molecular\ Weight_{cytokine} = 21\ kg/mole$

References

1. Watanabe T, Kubota S, Nagaya M, Ozaki S, Nagafuchi H, Akashi K, Taira Y, Tsukikawa S, Oowada S, Nakano S. The role of HMGB-1 on the development of necrosis during hepatic ischemia and hepatic ischemia/reperfusion injury in mice. *J Surg Res* (2005) **124**:59–66. doi:10.1016/j.jss.2004.10.019
2. Baumann A, Buchwald D, Annecke T, Hellmich M, Zahn PK, Hohn A. RECCAS -REmoval of Cytokines during CArdiac Surgery: study protocol for a randomised controlled trial. *Trials* (2016)1–8. doi:10.1186/s13063-016-1265-9
3. Tibi L, Chhabra SC, Sweeting VM, Winney RJ, Smith AF. Multiple forms of alkaline phosphatase in plasma of hemodialysis patients. *Clin Chem* (1991) **37**: Available at: http://clinchem.aaccjnls.org/content/37/6/815.long [Accessed July 29, 2017]
4. Su B, Zhou W, Dorman KS, Jones DE. Mathematical Modelling of Immune Response in Tissues. *Comput Math Methods Med* (2009) **10**:9–38. doi:10.1080/17486700801982713
5. Pigozzo AB, Macedo GC, Santos RW dos, Lobosco M. On the computational modeling of the innate immune system. *BMC Bioinformatics* (2013) **14 Suppl 6**:S7. doi:10.1186/1471-2105-14-S6-S7
6. Price CP. Multiple Forms of Human Serum Alkaline Phosphatase: Detection and Quantitation.

Ann Clin Biochem (1993) **30**:355–372. doi:10.1177/000456329303000403
7. Kiffer-Moreira T, Sheen CR, Gasque KCDS, Bolean M, Ciancaglini P, Van Elsas A, Hoylaerts MF, Millán JL. Catalytic signature of a heat-stable, chimeric human alkaline phosphatase with therapeutic potential. *PLoS One* (2014) **9**: doi:10.1371/journal.pone.0089374
8. Damas P, Ledoux D, Nys M, Vrindts Y, De Groote D, Franchimont P, Lamy M. Cytokine serum level during severe sepsis in human IL-6 as a marker of severity. *Ann Surg* (1992) **215**:356–362. doi:10.1097/00000658-199204000-00009
9. Kawai S, Sakayori S, Watanabe H, Nakagawa T, Inoue G, Kobayashi H. The Role of Interleukin-10 in Systemic Inflammatory Response Syndrome with Sepsis. *J Infect Chemother* (1998) **4**:121–127. doi:10.1007/BF02491513
10. Starr C, Taggart R. *Biology: The Unity and Diversity of Life*. (2009).
11. Nocedal J, Wright SJ. *Numerical optimization*. (2006). doi:10.1007/978-0-387-40065-5
12. Kawai S, Sakayori S, Watanabe H, Nakagawa T, Inoue G, Kobayashi H. The role of interleukin-10 in systemic inflammatory response syndrome with sepsis. *J Infect Chemother* (1998) **4**:121–127. doi:10.1007/BF02491513
13. Li Y, Karlin A, Loike JD, Silverstein SC. A critical concentration of neutrophils is required for effective bacterial killing in suspension. *Proc Natl Acad Sci U S A* (2002) **99**:8289–8294. doi:10.1073/pnas.122244799
14. Swameye I, Muller TG, Timmer J, Sandra O, Klingmuller U. Identification of nucleocytoplasmic cycling as a remote sensor in cellular signaling by databased modeling. *Proc Natl Acad Sci U S A* (2003) **100**:1028–1033. doi:10.1073/pnas.0237333100
15. Raue A, Kreutz C, Maiwald T, Bachmann J, Schilling M, Klingmüller U, Timmer J. Structural and practical identifiability analysis of partially observed dynamical models by exploiting the profile likelihood. *Bioinformatics* (2009) **25**:1923–1929. doi:10.1093/bioinformatics/btp358
16. Saltelli A, Chan K, Scott EM. *Sensitivity Analysis*. (2000). Available at: http://www.wiley.com/
17. Schaibly JH, Shuler KE. Study of the sensitivity of coupled reaction systems to uncertainties in rate coefficients. II Applications. *J Chem Phys* (1973) **59**:3879–3888. doi:10.1063/1.1680572
18. Cukier RI, Schaibly JH, Shuler KE. Study of the sensitivity of coupled reaction systems to uncertainties in rate coefficients. III. Analysis of the approximations. *J Chem Phys* (1975) **63**:1140–1149. doi:10.1063/1.431440
19. Cukier RI, Fortuin CM, Shuler KE, Petschek AG, Schaibly JH. Study of the sensitivity of coupled reaction systems to uncertainties in rate coefficients. I Theory. *J Chem Phys* (1973) **59**:3873–3878. doi:10.1063/1.1680571
20. Cukier RI, Levine HB, Shuler KE. Nonlinear sensitivity analysis of multiparameter model systems. in *Journal of Physical Chemistry*, 2365–2366. doi:10.1021/j100540a010
